# Targeting Aberrant RAS/RAF/MEK/ERK Signaling for Cancer Therapy

**DOI:** 10.3390/cells9010198

**Published:** 2020-01-13

**Authors:** Ufuk Degirmenci, Mei Wang, Jiancheng Hu

**Affiliations:** 1 Division of Cellular and Molecular Research, National Cancer Centre Singapore, 11 Hospital Crescent, Singapore 169610, Singapore; 2 Cancer and Stem Cell Biology Program, Duke-NUS Medical School, 8 College Road, Singapore 169857, Singapore

**Keywords:** RAS GTPases, RAF family kinases, Ras/RAF/MEK/ERK signaling, BRAF(V600E), RAF inhibitors, paradoxical activation, protein–protein interactions, synthetic lethal, neoplasm

## Abstract

The RAS/RAF/MEK/ERK (MAPK) signaling cascade is essential for cell inter- and intra-cellular communication, which regulates fundamental cell functions such as growth, survival, and differentiation. The MAPK pathway also integrates signals from complex intracellular networks in performing cellular functions. Despite the initial discovery of the core elements of the MAPK pathways nearly four decades ago, additional findings continue to make a thorough understanding of the molecular mechanisms involved in the regulation of this pathway challenging. Considerable effort has been focused on the regulation of RAF, especially after the discovery of drug resistance and paradoxical activation upon inhibitor binding to the kinase. RAF activity is regulated by phosphorylation and conformation-dependent regulation, including auto-inhibition and dimerization. In this review, we summarize the recent major findings in the study of the RAS/RAF/MEK/ERK signaling cascade, particularly with respect to the impact on clinical cancer therapy.

## 1. A Brief History of RAS/RAF/MEK/ERK Signaling Cascade

The period from 1964 to the 1980s was the era of oncogene discoveries; prominent viral oncogenes such as Ras (rat sarcoma) and Raf (rapidly accelerated fibrosarcoma) were identified during this time [1,2,3]. Shortly after their discovery, these viral oncogenes were shown to be altered versions of normal cellular genes [2,4,5,6]. Three cellular Ras genes encode the four members of the RAS family of small GTPases, KRAS4A, KRAS4B, HRAS, and NRAS, whose mutations drive one-third of human cancers. Instead of point mutations, V-RAF is an N-terminal truncated version of the cellular RAF gene (CRAF) that encodes a serine/threonine kinase [7,8]. These milestone discoveries instigated our current understanding of the dominant cancer signaling pathway: RAS/RAF/MEK/ERK. 

**Figure 1:**
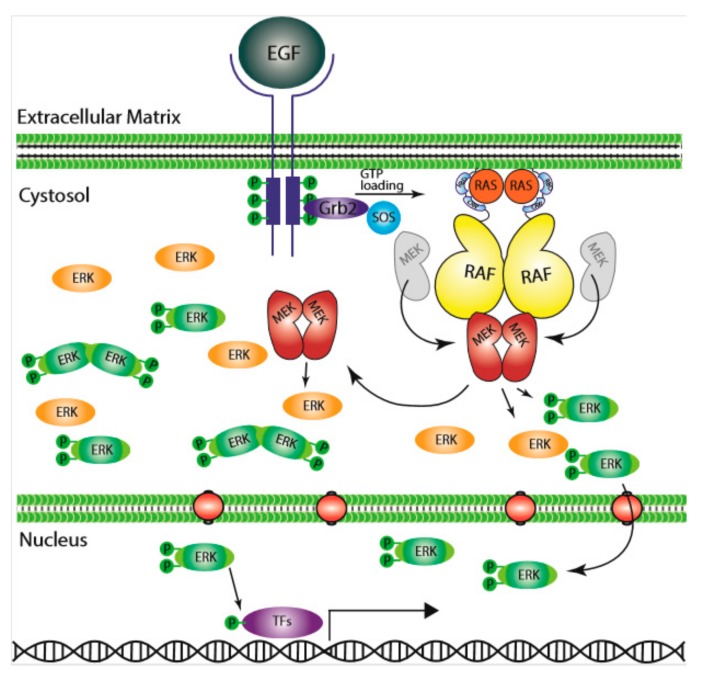
The Ras/RAF/MEK/ERK signaling pathway. Epidermal growth factor (EGF) initiates the signal on the cell surface through the EGF receptor (EGFR) (receptor tyrosine kinase), which activates guanine exchange factor to load RAS with GTP. RAS–GTP dimers/nanoclusters recruit RAFs or RAF/MEK heterodimers to plasma membranes, where RAF and MEK assemble transient tetramers that facilitate RAF activation through a back-to-back dimerization. MEKs docking on active RAF dimers further form face-to-face homodimers that are turned on by RAF. Activated MEKs phosphorylate ERKs, which generate response to the signal. CRR; Cys-rich region, RBD; Ras-binding domain.

In 1984, it was first suggested that epidermal growth factor (EGF) stimulated activation of RAS, i.e., increased its GTP bound state [9], which linked RAS to the upstream receptor tyrosine kinase (RTK) signaling. The subsequent finding of RAS requirement for serum-stimulated DNA replication further solidified its role at the plasma membrane as a signal transducer [10,11]. In the effort to unravel the function of RAF proteins, an early study showing that v-RAF could stimulate S-phase entry in the absence of RAS activity [11] suggested that RAF functions as either downstream of RAS or in parallel to RAS to promote cell division. Studies of RAF in *Drosophila* [12] and *C. elegans* [13] confirmed its role in RTK signaling, which put RAF under RTKs and RAS. In separate studies, the cytoplasmic Ser/Thr kinases ERK1 and ERK2 were found to promote cell cycling [14,15,16,17]; and ERK1/2 activity was shown to be enhanced by yet other cytosolic kinases, MEK1/2, that phosphorylate the conserved Thr/Tyr in the activation loop of ERK1/2 [18]. Further investigation of the kinase cascade revealed that CRAF is the upstream kinase that phosphorylates MEK1 at Ser222 and MEK2 at Ser218 that regulates the activity of MEK, and through which ERK [19,20], thus rank-ordering the MAPK signaling from RAS, RAF, MEK, and finally to ERK [21]. The RAS GTPase is “switched on” to the GTP-bound active form by upstream regulators, such as RTKs, activated Ras can then physically interact with RAF and turn on the signaling cascade [22,23,24,25]. These findings delineated how signals generated from membrane-bound receptors are conveyed through RAS GTPase and transmitted intracellularly through a kinase cascade, setting a milestone in understanding of cell communication and signaling (Figure 1). 

For RAF research, the early spotlight on CRAF was shifted to BRAF after the discovery in 2002 that BRAF mutations (especially BRAFV600E) were dominant in cancer [26]. Recent studies, however, have brought CRAF back to the center stage for its role in the complicated regulation of RAF kinases by the so called inhibitor-induced paradoxical activation of RAF seen in RAF and RAS mutant cancers [27]. A main therapeutic challenge in treating RAS/RAF-driven cancers is to develop drugs that can inhibit this pathway without paradoxical activation. 

There are several major reviews in the field that describe the importance of RAS and RAF signaling and their roles in cellular regulatory processes. In this paper, we refer to these reviews, at times, due to the abundance of original research articles. However, we do provide short summaries of crucial aspects of the field, with their primary references, where we feel it enhances the clarity of this review.

## 2. RAS GTPases and Their Activation

The mammalian RAS GTPases consist of three gene isoforms, HRAS, NRAS, and KRAS, and four protein isoforms (splicing isoforms of KRAS give rise to KRAS4A and KRAS4B proteins). Whilst the isoforms share most of their sequence, substantial differences appear in the C-terminal so-called hypervariable regions and in post-translational modifications [28,29,30]. These differences in sequence and modification are considered to underlie the findings that RAS isoforms can function differentially in different biology and pathophysiology [31,32,33,34,35]. From the standpoint of engaging MAPK signaling, KRAS is more efficient than HRAS for CRAF activation while the opposite is true for PI3K activation [36]. Furthermore, both KRAS and HRAS have higher activity toward NFκB activation in contrast to NRAS [37]. While being members of the most frequently mutated oncogene family in human cancer [38], RAS isoform mutants are clearly not equally prevalent in cancers [30]. KRAS mutations are overwhelmingly represented in cancers as whole compared to the other two isoforms. There is also strong tissue predilection of the occurrence of RAS isoform mutations; while KRAS monopolizes pancreatic cancers, NRAS mutants dominate melanoma and AML. Furthermore, the RAS isoforms also have different favored mutations, which interplay with cancer types and tissue origins [38], adding complexity and intrigue [31]. All these differences among RAS isoforms underscore the limitation of our understanding of RAS proteins and their downstream pathway engagements [33]. 

The cellular activities and functions of RAS proteins are regulated at different levels. As a GTPase, RAS activity is regulated by the GTP/GDP cycle [39]. GTP-bound RAS proteins adopt the so-called active conformation that allows them to bind and activate downstream effectors, while the GDP-bound RAS proteins have altered conformations that impede such interactions. The process of dislodging bound GDP for GTP, thereby activating RAS, is facilitated by guanine exchange factor (GEF) proteins. The intrinsic GTPase activity, enhanced by RAS GTPase activating protein (GAP) [40], hydrolyses the bound GTP into GDP and returns the protein to the inactive GDP-bound state, thus completing the GTP/GDP cycle. As an important signaling process, the GTP/GDP cycle is regulated by various stimuli, including the cell surface receptors, such as several RTKs. Genetic alterations that affect the regulation of RAS activation/inactivation cycle, particularly ones that result in the persistent activation of RAS, can lead to human pathologies—the so called RASopathies [41]. For example, upregulation or gain-of-function mutations of RTKs stimulate the activity of the RasGEF, Sos [42], which in turn elevates the cellular level of GTP-bound Ras and oncogenic transformation. On the other hand, the loss-of-function mutations of RasGAPs, exemplified by NF1 [43], also results in persistent RAS activation and proliferative diseases. The most common mutations leading to RAS activation are on RAS itself, which occur in one-third of human malignancies. There are two hot spots of RAS activating mutations: the mutations at glycines 12 and 13 (G12/13) that impair RAS association with RasGAPs and at glutamine 61 (Q61) that diminish the intrinsic GTPase activity of Ras [38].

The proper functions of RAS proteins are also subject to the regulation of their posttranslational modifications, which are essential for their trafficking, membrane localization, and interaction with regulators and effectors [28,44,45,46,47,48]. RAS proteins belong to group of proteins that contain the C-terminal CAAX consensus sequence for the prenylation processing [48,49]. The nascent RAS proteins in the cytosol are firstly modified by either protein farnesyltransferase or geranylgeranyltransferase on the cysteine residue of their CAAX box [44,50,51], after which they transiently associate with the endoplasmic reticulum (ER) [51,52,53]. On the ER, they are further processed by the endoprotease RCE1 [54], followed by isoprenylcysteine carboxylmethyltransferase ICMT [55], which converts the carboxyl-terminus of RAS proteins from a hydrophilic region into a hydrophobic one, facilitating the insertion of Ras proteins into cellular membranes [56,57]. Subsequently, RAS proteins can undergo isoform-specific modifications, such as phosphorylation for KRAS4B and palmitoylation for HRAS and NRAS, which facilitate transport and plasma membrane localization through distinct mechanisms [29]. 

Interestingly, the functions of RAS are subject to another layer of regulation—dimerization and nano-clustering on plasma membrane to trigger and transmit signaling downstream [58]. Lipid rafts, subdomains of plasma membranes that have distinct chemical composition and properties, have been known since 1998 [59] and were observed as lateral heterogeneity, and consequently non-random distribution, of proteins by proteolipid-based sorting. Earlier works in Ras nano-clustering showed that Ras proteins gather on lipid rafts differentially among isoforms [60,61]. The current model is that all Ras isoforms in their GDP and GTP bound state give rise to distinct conformations and interact with distinct lipid compositions, cholesterol, PS, PA, PIP_2_, PIP_3_, PI_3_P, and PI_4_P, and that lipid composition contributes to the stability of the nanoclusters of Ras [62]. RAS dimerization or nano-clustering is thought to be a key step in the generation of its ability to couple to RAF [61,62,63]. 

## 3. RAF Isoforms

**Figure 2:**
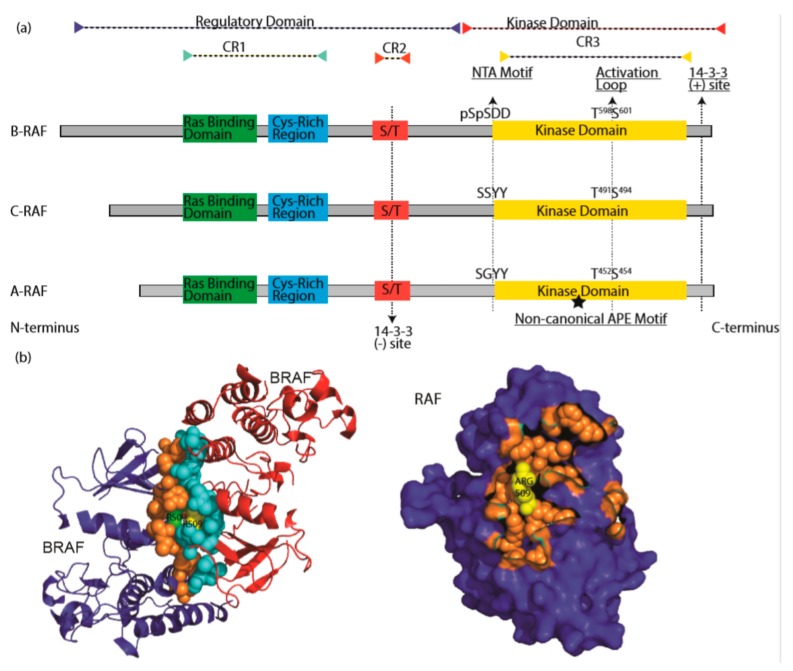
The RAF family kinases. (**a**) Conserved domains on three RAF proteins are shown in panel (**a**). CR1 contains a Ras binding domain and a Cys-rich region while CR2 includes a S/T phosphorylation site. The 14-3-3 binding at this region inhibits RAF. CR3 contains a putative kinase domain adjacent to an acidic N-terminus (NTA) and a regulatory C-terminus. At the C terminus, there is a secondary 14-3-3 binding site which promotes dimerization. The non-canonical APE motif of ARAF is labeled with a star. ((**b**), left) Dimer interface of BRAF is shown, crystallography data was obtained from [86], PDB ID: 4MNE. Blue and red color indicates two separate BRAF molecules. Orange (Blue BRAF) and turquoise colored (Red BRAF) sphere-shaped amino acids indicate the dimer interface. While R509 from both RAF molecule located at the center of the dimer interface, green R509 belongs to red BRAF, while yellow R509 does to blue BRAF. ((**b**), right) Crystal structure of dimer interface from plane of interaction with only blue BRAF chain is visible. Orange spheres indicate the amino acids at the dimer interface, with exception of Arg509, which is labeled with yellow color. Structure was drawn by using pyMOL. CR1: conserved region 1; CR2: conserved region 2; CR3: conserved region 3.

RAF proteins, pivotal components of Ras/RAF/MEK/ERK signaling cascade, include three isoforms: CRAF, BRAF, and ARAF. CRAF (also called Raf-1) is the first RAF protein identified in 1984, followed by ARAF [64,65] in 1986 and BRAF [66] in 1988. All RAF proteins share three conserved regions: CR1 (conserved region 1) [67,68], which contains a RAS-binding domain [69,70,71,72] (RBD) and a Cys-rich domain [73]; CR2, which is characterized by Ser/Thr-rich sequence; and CR3, which is constituted of a putative kinase domain with an acidic N-terminus (NTA) [74,75] and a regulatory C-terminus [76,77]. Structures of RAF kinases revealed that the proteins could be divided into two functional regions as the regulatory domain (CR1 and CR2) and kinase domain (CR3) [78,79]. Although they have similar molecular structures, RAF proteins have quite different activity and play differential roles in cell function. BRAF, which is well-known in cancer, as it is a major target of genetic mutations in tumorigenesis, has the highest activity among three isoforms, likely by virtue of its constitutively-phosphorylated NTA motif [75,80]. CRAF, which plays an indispensable role in RASopathies [81,82,83], has intermediate activity. Lastly, ARAF is rarely seen genetically altered and has the lowest kinase activity due, for the most part, to its non-canonical APE motif [84,85] (Figure 2a). 

As typical protein kinases, RAFs contain a kinase domain that comprises an N-lobe (amino-terminal lobe) and a larger C-lobe (Carboxyl-terminal lobe). The N-lobe that includes five antiparallel β-strands and a signal-integrated helix denoted as αC is connected by a flexible hinge to the C-lobe that mostly consists of α-helices and a key loop termed activation segment/activation loop. Although the kinase domains oscillate between closed and open conformations via inter-lobe motions, the active state is restricted to the closed conformation. The closed conformation is maintained by the alignment of two parallel hydrophobic cores (spines) of spatially conserved residues spanning N- and C-lobes [87,88]. The regulatory spine (R-spine) is built upon the alignment of four residues in RAFs, F516 from β4 strand, L505 from αC, F595 from DFG, and H574 from HRD (BRAF residue numbers used here) [80,89,90]. The catalytic spine (C-spine) is formed to control kinase activation upon ATP loading. These hydrophobic spines limit the movement of αC helix and stabilize the active conformation of the kinase domain [91,92]. 

In inactive conformation of RAF, the “Asp-Phe-Gly (DFG)” motif at the N terminus of the activation loop is flipped “OUT” relative to its conformation in the active state “IN”. The “OUT” DFG blocks the ATP binding pocket and stabilizes an open inactive conformation [93]. The αC helix adjacent to N-terminus of the dimer interface is also in an “OUT” position in the inactive kinase, where it binds to dimer interface residues (504–511 in BRAF) and further stabilizes the inactive conformation [87,94]. In addition, F485 from the β3 strand, L597 on the activation loop, and V600 on helix AS-H1 form a hydrophobic network that provides further stabilization of inactive conformation [95]. In the active state, αC assumes the “IN” conformation that helps the formation of a salt bridge between glutamine from αC and catalytic lysine from VAIK motif (E501-K483 for B-Raf, E393-K375 for C-Raf, and E354-K336 for A-Raf) [96]. The activation loop AS-H1 is disordered in the active conformation due to αC being pulled “IN”, which reciprocally enables dimerization [95]. It is important to note that the hallmark of an active RAF kinase requires the assembly of the two hydrophobic spines in its core, which supersedes the more dynamic/oscillating elements such as the so-called “open” or “closed” states or formation of the conserved salt bridge [88]. 

## 4. RAF-Knockout Mouse Models

Gene-knockout mouse models of the three RAF proteins have yielded a vast amount of information on their functional differences. CRAF-knockout mice are embryonic lethal with poor development of the placenta, liver, and hematopoietic organs [97,98]; BRAF-knockout mice die in utero at D12.5 with vascular and neuronal defects [99,100]. In contrast, ARAF-knockout mice were born alive, but presented severe intestinal and neurological abnormalities [101].

Interestingly, mice that express a kinase-impaired CRAF with the loss of phosphorylation sites in NTA motif (YY340/341FF) exhibit a different phenotype from that caused by complete CRAF-deficiency [102]. Several mechanisms have been suggested to explain potential kinase-independent functions of CRAF. Firstly, Rok-α is hyperactivated and mis-localized to the membrane in CRAF^−/−^ cells, which blocks Fas death receptor internalization, leading to increased Fas on the plasma membrane and Fas-dependent apoptosis [103,104]. Furthermore, CRAF NTA mutant mice show impaired wound healing and migration of keratinocytes [104]. CRAF-deficient fibroblasts and keratinocytes exhibit rounded morphology and impaired migration, indicating the defective cytoskeleton. This phenotype could be rescued with Rok-α double knockout. Biochemical and functional evidence support a mechanism for these phenotypes of kinase-independent actions of CRAF being that the autoinhibitory Cysteine-rich domain of CRAF acts in trans to inhibit Rok-α. This involvement of CRAF in the regulation of apoptosis has been shown to be relevant in several human diseases. For example, Rok-α inhibition by CRAF is a prerequisite for Ras-induced cellular transformation [81,82,105,106,107], and it has been shown that CRAF plays a role in pathogen-mediated macrophage apoptosis and erythroid differentiation [108,109].

ERK signaling can inhibit apoptosis in various ways, including expression of caspase inhibitors, neutralization of Bcl-2 family proteins [110,111], and activation of NFκB [112,113,114]. It is worth noting, however, that the anti-apoptotic role of CRAF does not depend on its ability to activate ERK. Additionally, targeting CRAF in KRAS G12V/Trp53 mutant lung tumors triggered massive apoptosis without blocking ERK phosphorylation, which may explain the acceptable levels of toxicity of this approach [107]. In addition to its role in regulating Fas, mitochondrial CRAF has been shown to protect the cell from apoptosis; indeed, some growth factors have been reported to play roles in the translocation of CRAF to mitochondria where p21-activated kinase (Pak1) phosphorylates it on Ser338 in the NTA motif [115,116,117]. There is also evidence suggesting that CRAF has a scaffold function for PKCθ to facilitate phosphorylation of BAD as a way of CRAF regulation [118]. In addition, CRAF directly interacts with voltage-dependent anion channels and prevents the release of cytochrome C from mitochondria [119]. Other targets of CRAF include two pro-apoptotic kinases, ASK1 [120,121,122] and MST2 [123], which also rely on a kinase-independent activity of CRAF. Deficiency of CRAF promotes apoptosis of cardiomyocyte, which can be rescued with ASK1 knockdown [124]. Despite these reports, the definitive mechanism through which mitochondrial CRAF prevents apoptosis requires further investigation. 

Similar to CRAF, ARAF also binds and sequesters MST2 independent of its kinase activity [125]. ARAF interaction with MST2 is dependent on the presence of full-length splicing product controlled by hnRNP H [126]. In primary tumors and cell lines, ARAF and MST2 co-localize to mitochondria and prevent apoptosis [126]. In addition to MST2, ARAF was found to bind pyruvate kinase M2 (PKM2), which is an embryonic splice variant of PKM and responsible for aerobic glycolysis [127]. In ARAF-transformed NIH3T3 cells, PKM2 is changed from dimeric to a highly active tetrameric conformation, linking ARAF to cancer metabolism [128]. 

Double knockout of RAF proteins generated more severe phenotype which suggested their additive effects [129,130]. Studies indicated that RAF proteins could compensate for lack of one of the isoforms up to a certain point but all three are required for regulating the development of the organism. In addition to knockouts, constitutively-active BRAFV600E mutant mouse also showed a embryonically lethal phenotype [131]. 

## 5. Activation of RAF Proteins

### 5.1. Auto-Inhibited State of RAF

Once the RAS/RAF/MEK/ERK pathway was identified, research efforts focused on studying both negative and positive regulators of the signaling cascade. In non-dividing cells, RAF exists in an auto-inhibited state where its N-terminus docks on the kinase domain, inhibiting its catalytic activity [132,133]. This is supported by the fact that overexpression of the N-terminal CR1 domain inhibits kinase activity in trans [132]. The interaction between the two parts of CRAF was further validated by the study of the N-terminus mutations of CRAF in regulating its kinase activity [132]. Moreover, RAF inhibitors that disrupt the auto-inhibited state trigger paradoxical activation, much to the disappointment of oncologists [134,135].

### 5.2. RAF Recruitment to Plasma Membrane by Activated RAS

RAS proteins are anchored on the plasma membrane through their prenylated C-terminal CAAX motif [136]. Upon GTP-loading, RAS is able to recruit RAF to the plasma membrane via its RAS-binding domain (RBD) [137], which consists of a ubiquitous fold shared by other RAS effectors such as PI3K p110 subunits and RAL guanine nucleotide dissociation stimulator (RALGDS) [69,138,139]. Single residue substitution in the RBD is sufficient to disrupt the association of RAF with RAS and abolish the activation of RAF [140]. Outside of the RBD, the Cys-rich region has been shown to form zinc coordinated structures that interact with phospholipids and facilitate membrane translocation of several kinases, including CRAF [141,142,143,144,145,146]. Furthermore, the Cys-rich region of RAF can also interact with the farnesyl group in RAS proteins [143,144,145,146,147]. In addition, the N-terminus before RBD domain (also called as N’-segment) has been shown to regulate the binding selectivity of both RAF and Ras isoforms [96,143,148]. Thus, RBD, Cys-rich region and N’-segment are involved in the plasma membrane recruitment of RAF proteins by active Ras. Despite the large amount of work and progress made in the understanding of RAS–RAF interaction, there are critical details missing on the major structural and functional interactions of these two proteins. For example, it is not clear how RAS triggers the de-phosphorylation of inhibitory Ser residues [149]. In addition, the manner through which the auto-inhibitory intramolecular interactions of RAF proteins are relieved is also not yet clear [150], although Phosphatase 2A (PP2A) [151] and PP1 [152,153] have been shown to regulate this process [150,151,152,153,154]. 

### 5.3. Dimerization Is a Key Event in RAF Activation

Despite the differences in their ability to trigger downstream ERK signaling, all RAF isoforms are activated through dimer-driven transactivation. Under physiological condition, Ras-driven activation of RAF proteins occurs on the plasma membrane where activated RAS promotes RAF dimerization, a key event to trigger the kinase activity of RAF proteins. The notion of dimerization-driven transactivation of RAF proteins arises from an early observation that artificial oligomerization of CRAF triggers its kinase activity [155,156]. The subsequent observation of RAS-induced heterodimerization of BRAF and CRAF under physiological conditions further supports the relevance of dimer formation [157,158]. The finding that kinase-dead BRAF was able to activate ERK signaling through dimerizing with and activating CRAF not only provide further support for the role of RAF dimerization [159], but raised the awareness that both catalysis-dependent and -independent functions of RAF are functionally important [158,160,161]. In addition to the BRAF–CRAF heterodimer, respective homodimers of the two isoforms, i.e., BRAF:BRAF and CRAF:CRAF, have also been detected, but were noted to have lower kinase activity [159]. ARAF, however, has the lowest affinity for dimer formation due to its different structural features, most notably its non-canonical APE motif that does not stabilize the dimer interface as BRAF and CRAF are able to do [84]. However, site-specific mutagenesis of this motif from AAE to APE enables ARAF to form dimers as strongly as CRAF. In summary, RAF family members can form physiologically relevant heterodimers and homodimers, resulting in their transactivation [88,162] (Figure 2b). 

The dimeric structure of RAF stabilizes the closed conformation of the kinase domain by limiting the oscillating motion of the two kinase lobes and promoting key conformational transitions [158]. Conversely, the acquisition of the active conformation also facilitates RAF dimerization. Once RAF achieves active conformation, its dimer interface becomes further stabilized by the hydrophobic R-spine residue in the αC- helix (L505 for B-Raf, L397 for C-Raf, and L358 for A-Raf) located adjacent to the conserved RKTR motif, which is allosterically connected αC-helix and dimer interface [163]. Upon the relocation of R509 to the center of the dimer interface, αC-helix interacts with the NTA motif of the trans RAF molecule and adopts the “IN” conformation [80,164] 

The dimerization of RAF proteins can be promoted by shortening their β3-αC loop; and in-frame deletions of β3-αC loop activate ARAF by enforcing homodimer formation, showing that ARAF can function as a “true” kinase to induce ERK phosphorylation [84], even though it is the weakest kinase in the family. The corresponding deletions in BRAF also ramp up its kinase activity through enhancing its homodimer formation. A surprising finding from these studies is that the kinase-dead BRAF mutant with an in-frame deletion of β3-αC loop (ΔNVTAP/V471F) can activate ERK signaling in the absence of active RAS [84] due to its substantial dimer affinity.

The dimerization of RAF proteins can be also improved in other ways. The BRAF splicing variants lacked exon 4–7 exhibit a stronger dimer affinity and thereby a strong resistance to RAF inhibitors [165]. In addition to alternative splicing products; gene fusions, translocations and deletions that remove the auto-inhibitory N-terminus allow RAFs to homodimerize with higher affinity [166,167,168,169]. Furthermore, RAF inhibitors, especially the first-generation drugs, enhance RAF dimerization upon their binding, and thereby paradoxically activate the pathway, which is further discussed below. Understanding the complete mechanism of dimerization is still a work in progress, with questions remaining about the scaffolding proteins, activation direction, and the priming of the cells for RTK signaling by increasing RAS nanoclusters. 

### 5.4. The Role of NTA Motif and Activation Loop Phosphorylation in RAF Activation

RAF proteins undergo multiple phosphorylation events during their activation cycle. Two major phosphorylation events, the NTA motif and the activation loop, play key roles. The NTA motif contains divergent loci for phosphorylation among RAFs, allowing them to have distinct regulations [75]. In BRAF, the NTA motif contains SSDD, in which the D448 and D449 provide the initial negative charge before serine phosphorylation. In CRAF, this locus contains SSYY and requires phosphorylation of both serine and tyrosine residues. The constitutively phosphorylated SS and acidic DD in the NTA motif of BRAF can explain the higher activity of BRAF compared to ARAF and CRAF. Negative charges in this loci contribute to the relief of autoinhibition [132,150,170] and are critical for dimerization-driven transactivation [80]. In RAF homo/heterodimers, the phosphorylated NTA motif was suggested by molecular modeling to form multiple salt bridges that extend and stabilize the dimer interface between two protomers [116,129]. It has been shown that the phosphorylation status of NTA motif dictates the direction of transactivation in RAF dimers [80]; the protomer with phosphorylated NTA motif acts as an activator, while the other protomer with non-phosphorylated NTA motif does as a receiver in RAF dimers. A ‘receiver’ protomer can be switched into an ‘activator’ protomer upon phosphorylation in its NTA and thereby amplify the dimerization-driven transactivation of RAF proteins. Protein kinases that target the NTA motif play an important role not only in the activation of RAF proteins but also by controlling the receiver–activator switch. SRC family kinases are the main kinase family targeting YY in ARAF and CRAF [171,172,173,174,175]. Although still controversial [176], p21-activated kinase (Pak) family members acting downstream of PI3K-CDC42 or RAC signaling cascades have been suggested to phosphorylate Ser338 [177,178] in CRAF. Other kinases that potentially target the NTA motif of RAF proteins include the following: (1) Janus Kinase 2 (JAK2), which is able to phosphorylate CRAF to activate MEK1 [179], (2) Casein Kinase 2 (CK2), which phosphorylates CRAF at Ser338 and BRAF at Ser446 [180], and (3) Calcium/calmodulin-dependent protein kinase II (CaMKII), which can phosphorylate CRAF at S338 [181].

The phosphorylation of activation loop is also important for the function of RAFs. The dimerization of RAF proteins facilitates their transition to an active conformation, which directly induces their activation loop auto-phosphorylation as a consequence. Like most protein kinases, the activation loop of RAFs contain two conserved phosphorylation sites relevant to their kinase function; for ARAF, these are Thr452 and Thr455 [182]; for BRAF, these are Thr599 and Ser602 [183]; and for CRAF, these are Thr491 and Ser494 [184]. Although studying the phosphorylation of the activation loop is difficult due to highly dynamic de-phosphorylation, the data from RAF co-activation assay suggest that cis auto-phosphorylation is the mechanism [80]. A recent study of a mouse model with BRAF T599A/S602A mutation [185,186] showed that the loss of activation loop phosphorylation could not duplicate the lethal phenotype of BRAF null mice. However, these mice had an aberrant development of the hematopoietic system and reduction of p-ERK level in the brain, among other characteristics, indicating the importance of proper activation loop phosphorylation of BRAF in development, function, and maintenance of cell populations [187]. 

## 6. Regulation of RAF by Accessory Molecules

Activation of RAF is also regulated by other proteins, including heat-shock protein 90(Hsp90) [188,189], CDC37 [188], kinase suppressor of Ras (KSR), and 14-3-3 [190,191,192,193] proteins. 

### 6.1. Hsp90/Cdc37 Chaperone Complex

The Hsp90/Cdc37 chaperone complex participates in proper protein folding that stabilizes protein kinases, including RAFs [194]. The association of RAF proteins with hsp90/cdc37 complex is essential for their activity towards MEK. Further, the association of BRAF with hsp90/cdc37 complex facilitates the assembly of high molecular weight BRAF complex and promotes its kinase activity, probably by increasing dimer affinity [195]. It is not surprising, therefore, that Hsp90 inhibitors can block the activity of RAF proteins and also induce their degradation, especially that of the constitutively active RAF mutants such as BRAF(V600E) [188,196,197]. Recently, Hsp90 inhibitors were shown to prevent development of resistance to RAF inhibitors on BRAF(V600E)-harboring cancers in clinical trials, even though the underlying mechanism were too complicated to pinpoint a single molecule [198,199,200]. 

### 6.2. KSR

Kinase suppressor of RAS (KSR) was initially identified through screening molecules essential for Ras function in Drosophila [201]. It had been referred to as pseudo-kinase due to its low kinase activity [202]. Subsequently, KSR was identified as a scaffold protein that not only brings close proximity between RAF and MEK1 [203], but also allosterically activates RAF [158,204,205,206]. It should be noted that KSR forms not only a side-to-side dimer with RAF proteins but also a face-to-face dimer with MEK, both of which are critical for its ability to transactivate RAF proteins [158,207,208]. 

### 6.3. Proteins 14-3-3

The 14-3-3 proteins were the first identified, and the most well-known, phosphoserine/phosphothreonine binding proteins, which can interact with a wide variety of proteins including transcriptions factors, cytoskeletal proteins, apoptosis factors and tumor suppressors. Binding to 14-3-3 can alter the proteins’ stability, localization, conformation, and association with other proteins. The functions of 14-3-3 has been reviewed extensively [209,210]. 

The 14-3-3 binds to phospho-serine/threonine residues in two conserved motifs of RAF proteins: RSXpS/TXP or RXXXpSXP [78,211]. RAF proteins contain two 14-3-3 binding sites: S259 and S621 for CRAF [79,212]; S365 and S729 for BRAF; and S214 and S582 for ARAF (Figure 2a). The 14-3-3 association with these two sites plays opposite roles in RAF activity. Activation of RAF by 14-3-3 occurs in the event of 14-3-3 binding two RAF molecules at the C-terminal phosphoserine, which promotes dimerization. Cryo-EM studies have shown that a dimeric 14-3-3 binds two phosphorylated serine residues on different RAF proteins, such as CRAF at Ser621 and BRAF at Ser 729, and thereby stabilizes the side-to-side heterodimer or homodimer [77,213]. On the other hand, if a dimeric 14-3-3 binds to the N- and C-terminus of a single RAF, respectively, it can stabilize autoinhibitory conformation [211,213]. The serine residues in conserved 14-3-3 binding motifs can be phosphorylated by Protein Kinase A (PKA) [214,215,216], Akt [216,217,218], AMPK [219,220], or by LATS1 from the MST2-Hippo pathway [221]. Regulation of phosphorylation events at two 14-3-3 binding sites can change the response drastically due to their opposite activity. The 14-3-3 binding site phosphorylation by different kinases may influence the therapeutic efficacy of cancer drugs. For example, in Ras-mutated cancer cells, the CRAF S621 is phosphorylated redundantly by AMPK and itself. Combination of RAF inhibitors with AMPK inhibitor could reduce the paradoxical activation [219]. Recognition of the alternative kinases that could phosphorylate 14-3-3 binding sites and thereby alter RAF activity, could improve clinical success. 

## 7. RAF Function as a Dimer

As mentioned above, the Arg at the center of RAF dimer interface is a key residue for RAF function, and its mutation to His blocks the dimerization-driven transactivation of RAF proteins [158]. This mutation can also abolish the drug resistance of BRAF(V600E) splicing variants that lack a part of N-terminus and thereby have a higher dimer affinity than their prototype [165]. Based on these observations, a monomer hypothesis in which RAF inhibitors bind and inhibit monomeric BRAF(V600E), but not dimeric variants, has been suggested to explain the drug resistance of BRAF mutants. However, this hypothesis has been challenged by other findings [80,84,95,195,222,223]. Firstly, it was shown that BRAF(V600E) has an extended dimer interface in contrast to its wild-type counterpart and exists as dimer/oligomer when expressed in cells [195,223]. Secondly, the Arg-to-His mutation did not fully diminish dimer formation of RAF proteins [223], and some RAF mutants with high dimer affinity such as BRAF(ΔNVTAP) and BRAF(ΔMLN) were still able to transactivate wild-type RAF in the presence of Arg-to-His mutation [84]. Thirdly, the Arg-to-His mutation, together with APE motif alteration, completely dissociated BRAF(V600E) dimers and abolished their activity, which could be rescued by GST fusion-mediated re-dimerization [84]. Lastly, RAF had been shown to phosphorylate MEK in a dimer-to-dimer manner in which an active RAF needs the other RAF partner to facilitate MEK phosphorylation (further discussed below) [84]. Therefore, the active monomer hypothesis is not supported at its current standing. 

## 8. RAF–MEK Heterodimerization and MEK–MEK Homodimerization, Essential Events for Signaling

MEK was identified independently by multiple groups as a substrate of RAF in 1992 [19,224,225], and their interaction was further supported by the yeast two-hybrid assay [226]. Following these findings, MEK was shown to be phosphorylated by RAF on Ser218 and Ser222 in its activation loop [227]. Recent studies have provided detailed mechanisms of RAF phosphorylation and activation of MEK. As a substrate, MEK needs to be recruited to RAF before activation. In quiescent cells, BRAF and MEK form a heterodimer in the cytosol, while CRAF and ARAF do not interact with MEK under this condition [86], presenting the question of how MEK is recruited to these RAFs. Crystallography studies have revealed that BRAF interacts with MEK1 in a face-to-face manner through two contact sites [86,208]. The first contact site is αG helices, a structural component of kinase–substrate docking interaction. The second contact site is consisting of their activation loop which generates antiparallel β-sheet [86,208]. Mutations on both contact sites that disrupt the BRAF/MEK interaction block both allosteric and catalytic activities of BRAF [84], suggesting that the RAF/MEK association plays an indispensable role in signal transmission from RAF to MEK. 

Given the abilities of RAF proteins to form side-to-side dimers with themselves and face-to-face dimers with MEK, it is not surprising that RAF and MEK assemble a tetramer complex of MEK:RAF:RAF:MEK in the process of activation, which has been captured in crystal structures [86]. Although how the RAF dimer phosphorylates MEK in this transient RAF/MEK tetramer is not completely understood, recent studies suggested that two MEK molecules need form a homodimer that is further phosphorylated by RAF dimer or itself [84,222], since monomeric MEK cannot be phosphorylated by RAF, and MEK homodimerization drives autophosphorylation. Moreover, phosphorylated MEK exerts its activity towards ERK as a dimer [222], suggesting that MEK/MEK homodimerization plays a critical role, as do RAF/RAF and RAF/MEK dimerizations, in the pathway activation.

## 9. Feedback Inhibition and Return to the Inactive State

Cessation of the activated ERK signal is a crucial part of controlled cell division; therefore, the triggered ERK pathway needs to return to the basal state with regulated feedback cues. As a part of immediate inhibitory phosphorylation; SOS1, the RAS-GEF, is phosphorylated by ERK, which inhibits interaction with Grb2 and creates 14-3-3 binding site for inhibitory binding [228,229]. ERK also exerts negative feedback on RAF by phosphorylating multiple Ser/Thr sites on RAF [230,231,232]. ERK phosphorylation of these Ser/Thr sites breaks the interaction of RAF with Ras, and also RAF-RAF dimerization [229]. Additionally, some of these Ser/Thr sites can also be phosphorylated by c-Jun amino-terminal kinases (JNKs) as a protective mechanism under stress conditions [233]. Further, the autophosphorylation of P-loop residues in RAF proteins serves as another feedback mechanism that impairs their kinase activity [234]. For MEK, it can be phosphorylated at its N-terminus that intercepts its activity [235].

Shortly after inhibitory phosphorylation as described above, the components of Ras/RAF/MEK/ERK signaling cascade require dephosphorylation to return to the inactive state. It has been shown that PP5 participates in the dephosphorylation of pSer338 in the NTA motif of CRAF [236], while PP2A is involved in Ser/Thr site dephosphorylation in a PIN1 dependent manner [231]. However, in the area of dephosphorylation, most underlying molecular mechanisms remain to be elucidated. 

Outside of the signaling cascade and immediate inhibitory phosphorylation, Dual-specificity phosphatases (DUSP) and Sprouty proteins are involved in transcriptional inhibition of the ERK signaling [237]. DUSP6 is upregulated by its transcription factor that activated by ERK1/2 [238,239]. Sprouty proteins are induced upon growth factor treatment [240], which can inhibit ERK signaling through different mechanisms. Spry1 and Spry2 are phosphorylated at their N-terminus upon RTK activation, which enables Spry to dock on Grb2 [241]. Furthermore, Spry4 inhibits ERK signaling by binding to CRAF [242]. 

## 10. Mutations in the Ras/RAF/MEK/ERK Signaling Cascade

The RAS signaling cascade has well-defined role in tumorigenesis. Point mutations across each of the members within the cascade have been identified as either driver of the tumor formation (RAS and RAF mutations) or indicator of poor prognosis (MEK and ERK mutations). Interestingly, driver mutations within the pathway are mutually exclusive. In this section, we describe the mutation hotspots for each member of the signaling cascade.

### 10.1. Ras Mutations

RAS mutations in all cancer types occur mostly in KRAS (85%), followed by NRAS (12%) and HRAS (3%). Interestingly, mutants of different RAS isoforms have tissue/cancer-type-dependent distributions. For example, NRAS is the most common in melanoma, while HRAS in adrenal glands and KRAS in pancreas [243]. Moreover, KRAS G12D was shown to promote stronger colon cancer development than NRAS G12D in Apc-deficient mice [244], and HRAS G12V knock into the KRAS locus was not tumorigenic in the lungs of mice [245]. 

**Figure 3:**
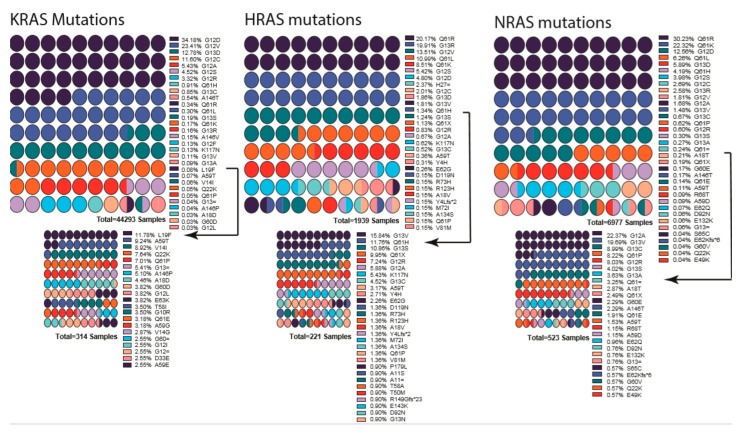
List of mutations detected in each RAS gene isoform. Percentages are indicated next to the mutation, and colors indicate the mutation. Low-percentage mutations were shown as a smaller graph underneath due to their being almost invisible in first graph. Arrows indicate from which point onward graphs were cut into two. Color scheme repeats every ten mutations and should be interpreted in combination of percentages and order. Graphs were drawn by using Prism 8.

RAS proteins have two mutational hotspots, 12–13 and 61. Most mutations changing G12 or G13, likely to intercept GAP’s Arg finger loop accession to the RAS GTPase site and prevent it from promoting hydrolysis [246,247,248]. As a consequence, G12 and G13 mutants trap RAS in a constitutively active state. Mutations at Q61 inhibits intrinsic GTP hydrolysis and GAP-mediated GTP hydrolysis (Figure 3) [246]. It is worth noting that, in addition to the isoform-dependent tissue distribution in RAS-driven cancers, the site of mutation hotspots also comes into play in the tissue/cancer-type distribution. For example, 90% of KRAS mutations in pancreatic ductal adenocarcinoma are at the position G12, while 90% of NRAS mutations in melanoma are at Q61. These patterns might indicate underlying fundamental signaling landscapes and RAS mutant interplay with these landscapes. Adding to the complexity of RAS mutations, the oncogenic stimuli also mold the site of mutation and the type of tumor development. For example, KRAS Q61R/L dominates the G12 mutation in urethane treated mice [31]. 

### 10.2. RAF Mutations

**Figure 4:**
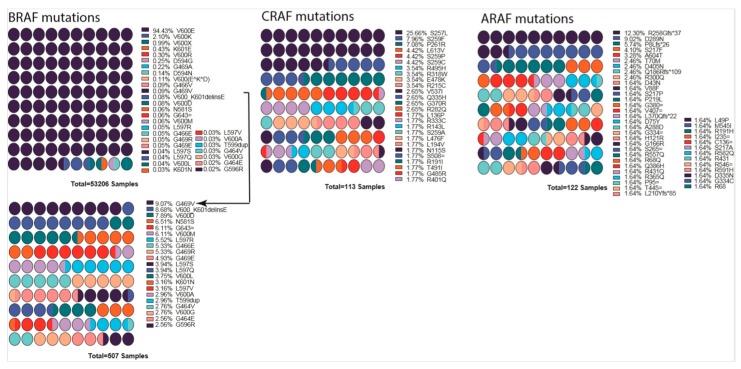
List of mutations detected in each RAF gene isoform. Percentages are indicated next to the mutation, and colors indicate the mutation. BRAF low percentage mutations are shown as a smaller graph underneath due to their being almost invisible in the first graph. Arrow indicates from which point onward graph was cut into two. Mutation data were obtained from COSMIC database. Color scheme repeats every ten mutations and should be interpreted in combination of percentages and order. Graphs were drawn by using Prism 8.

Although V-RAF was discovered as a first oncogenic Ser/Thr protein kinase, its cellular prototype CRAF is rarely mutated in cancers. The function of RAF as a prominent cancer driver was not established until the discovery of BRAF(V600E) in 2002 [26,249]. In cancer genomes, BRAF is a major target of oncogenic mutations and a single-point mutation, and V600E represents >90% of events, while mutations in CRAF, ARAF, and KSRs are much less and have been detected in decreasing order of frequency (Figure 4) [250]. 

Cancer-related mutations of RAF are enriched in special domains of the proteins and can be categorized into multiple groups based on how they trigger the pathway. The first group (or Class I) of RAF mutations activate RAF by mimicking phosphorylation of the activation loop. The second group (or Class II) of RAF mutations turn on RAF activity by relieving the auto-inhibitory status. The third group (or Class III) of RAF mutations have no, or impaired, kinase activity and agonist the pathway through transactivating their wild-type counterparts. 

Class I RAF mutations include V600E/D mutations in activation loop of BRAF and constitute the largest group in the RAF mutation spectrum [251]. The V600E/D mutation stabilizes the active conformation of BRAF by forming a salt bridge with K507 [252,253], thereby dramatically triggering the kinase activity of BRAF independent of Ras [157,165,223,231]. Class II RAF mutations are mainly located at the activation loop (K601 [254] and L597 [255,256]), or Gly-rich loop (P-loop, G464 [257] and G469 [258]), and disrupt the inhibitory interaction of activation loop with Gly-rich loop and thus destabilize the auto-inhibitory status [253]. This type of mutant has intermediate kinase activity and increased dimer affinity, and it triggers ERK signaling with or without active RAS. Class III RAF mutations are mostly found in the Gly-rich loop (G466 [257,259] and G469E [260]), the DFG motif (D594 [254,261] and G596 [254,259]), the catalytic loop (N581 [252]), or the C-spine (V471F [91]). These mutants have greatly reduced kinase activity compared with wild-type RAF and drive the activation of ERK signaling by transactivating the wild-type RAF with their enhanced dimerization affinity [160,252,262]. Unlike the Class I and Class II mutants, the Class III mutants require active Ras to trigger signaling cascade [80,262].

In addition to the highly prevalent mutations above, there are also some minor populations of RAF mutations in cancer genomes. N-terminal truncations of CR1 and CR2 domains, or kinase domain fusions with other proteins that relieve the N-terminal inhibition, have been identified in RAF genes [167,169,263,264,265]. These N-terminal truncations or kinase domain fusions allow RAF to dimerize and activate ERK signaling in a RAS-independent manner. Under physiological conditions, RAF dimerization occurs on the plasma membrane. The mutations in the Cysteine-rich domain and Ras-binding domain (RBD) of RAF can promote its plasma membrane recruitment, and thereby dimerization, to trigger ERK signaling [250,266,267,268,269]. In addition, mutations that abolish the inhibitory 14-3-3 association with the N-terminus of RAF (S259 in CRAF and S365 in BRAF) were also found in a small group of cancers [149,186,270,271].

### 10.3. MEK and ERK Mutations

In contrast to Ras and RAF mutations, MEK mutations are much less common in cancer genomes. These mutations do not co-occur with Ras or RAF mutations, indicating that they function as cancer drivers [272]. MEK mutations are classified into two groups according to their activation mechanism. The first group of MEK mutations turns on the kinase activity of MEK by disrupting the inhibitory intramolecular interaction mediated by the regulatory helix A, while the second group does so through enhancing MEK homodimerization. These two types of MEK mutants also exhibit differential sensitivities to MEK inhibitors in clinic or under clinic trials [222]. Like RAF, the elevated dimer affinity may also result in the resistance to inhibitors. Finally, ERK mutations are very rare in cancer genomes. However, a mutational hotspot has been identified on the site of D321/E322, which blocks the Dusp6-mediated dephosphorylation of ERK and thereby extends the half-life of active ERK [273].

## 11. Targeted Therapies against Hyperactive Ras/RAF/MEK/ERK Signaling in Cancers: The Present State and Perspectives

### 11.1. Is RAS Druggable?

Oncogenic Ras mutants have been considered “undruggable” over the decades due to their high affinity with GTP and the lack of proper binding pocket for small molecule inhibitor binding [274]. Recently, the Shokat group at UCSF showed that KRAS G12C could be targeted by using a covalent small molecule that docks in the switch II pocket and cross-links with Cys12 [275]. This discovery spurred the race to develop KRAS-G12C-targeting drugs for clinical use. AMG510 and MRTX1257 were the first ones to be developed as drugs to target KRAS G12C mutant for non-small cell lung cancer [274,276,277,278]. The results from phase I trials were promising and showed a 50% response rate for patients with KRAS G12C mutations. However, these drugs still require further investigation; besides validating efficacy, the impact of co-mutations and the development of drug resistance also need to be evaluated [276]. Unfortunately, KRAS G12C is only represented in a small fraction of RAS mutant cancers, and the challenge remains to drug the other RAS mutants. 

Among alternatives to target mutant RAS isoforms, inhibiting their functionally relevant post-translational modification represents a promising approach. The protein prenylation processing pathway attracted significant attention in the early targeted therapeutic field [48,279]. Initial effort was largely focused on protein farnesyltransferase, for good reason [48,279]. First, it was discovered as the canonical prenylation enzyme for all RAS isoforms under normal cellular conditions [44,51]. Second, it has been under careful genetic, biochemical, structural and functional studies [280,281,282,283]. However, as the first targeted therapy development, inadequate understanding of the alternative prenylation pathway of K- and N-RAS by geranylgeranyl transferase and corresponding inadequate stratification method led to the dismal clinical results for farnesyltransferase inhibitors. In recent years, new trials have been initiated that are designed to target the HRAS driven cancers, as H-RAS is not subject to alternate prenylation [284]. It will be interesting to see the level of efficacy from these trials; early data has shown promise (https://www.aacr.org/Newsroom/Pages/News-Release-Detail.aspx?ItemID=1350).

The earlier dismal trial results for farnesyltransferase inhibitors also sparked the interest in protein geranylgeranyl transferase (GGTase I), which prenylates K- and NRAS when farnesyltransferase is inhibited [284,285]. Targeting GGTase I, alone or in combination with FTIs, became an active area of investigation following the FTI trials [286]. Indeed, there are major efforts in developing GGTase I inhibitors for the treatment of certain cancers [286,287,288]. However, the toxicity of combination therapy of GGTase I and FTase may have limited the pace of such development [289]. Among the three steps of prenylation modifications, the last step of carboxymethylation by isoprenylcysteine carboxylmethyltransferase (ICMT) has also attracted much attention. Genetic suppression of ICMT in both cell and mouse models showed that ICMT inhibition led to the inhibition of tumorigenesis in multiple cancer model systems [55,290]. Proof-of-concept ICMT inhibitors have largely replicated the efficacy of genetic cancer models [291,292,293]. It will be quite interesting to follow the progress and evolvement of the development. 

For some RAS mutants, targeting their key effectors could be an effective alternative method. The small molecule inhibitor Rigosertib has been used to block the interactions of active Ras with the Ras-binding domain of effectors, and it has shown efficacy to target Ras-mutated cancers in phase III clinic trials [294]. Sulindac and MCP110, two other Ras-effector interaction inhibitors, were also shown to inhibit RAS driven tumorigenesis [295,296,297].

Like other oncogenes, Ras mutants need a set of cellular factors to facilitate their oncogenicity. These synthetic lethal factors/networks could be targeted for cancer therapy. Two recent studies showed that targeting RAF/MEK/ERK signaling together with autophagy signaling led to synthetic lethality and achieved a promising efficacy on Ras-driven cancers [298,299], suggesting that synthetic lethal factors/networks may be highly valuable for developing new drugs/methods against Ras-mutated cancers. Indeed, prior evidence of manipulating autophagy by inhibiting ICMT has shown encouraging results [300,301,302], which supports that ICMT can be used to regulate RAS-driven signaling. For the effort of identifying the targets downstream of ERK signaling in modulating autophagy, several screens have been carried out with whole genome shRNA knockdown libraries, which yielded few druggable factors [303]. The strong biology, however, appeals to future reliable screening approaches such as Cas9-CRISPR-mediated knockout [304]. Nevertheless, exploring all synthetic lethal factors/networks of Ras should provide better understanding how oncogenic Ras induces cancers and also accelerate the development of drugs against Ras-mutated cancers.

### 11.2. RAF/MEK/ERK Inhibitors and Resistance

**Figure 5:**
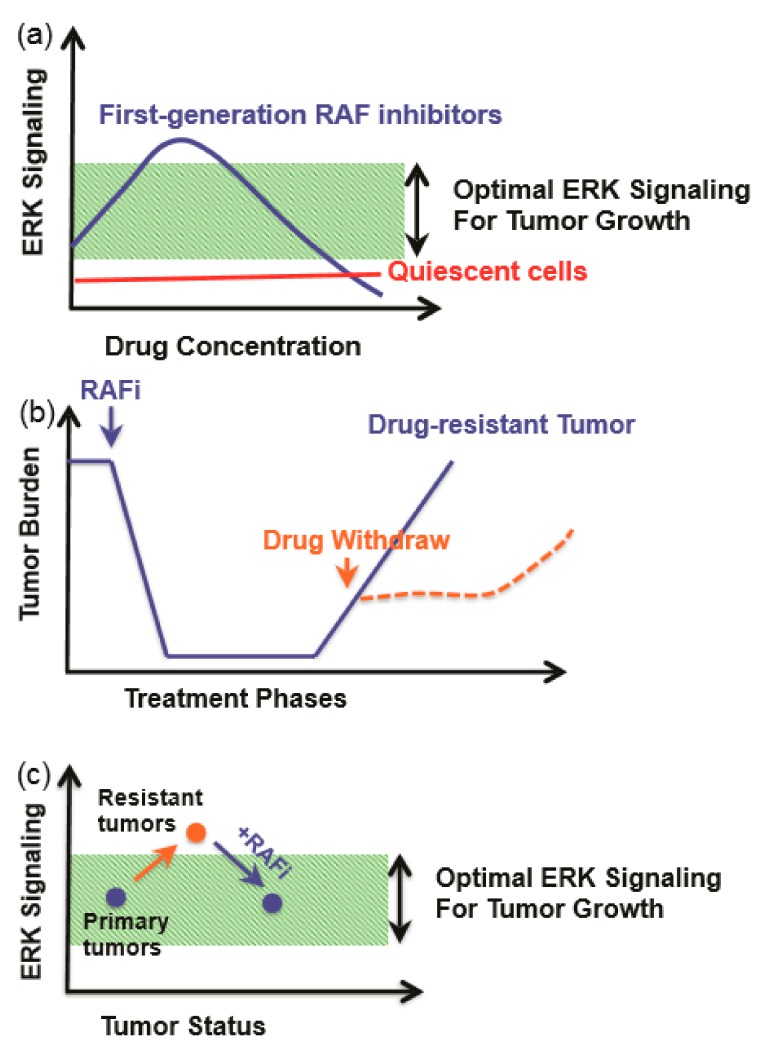
An optimal ERK signaling is required for tumor growth. (**a**) First-generation RAF inhibitors paradoxically agonist ERK signaling in cancer cells with active Ras. (**b**) Drug-resistant tumors rely on the presence of the drug for optimal growth, and a removal of the drug delays tumor progression. (**c**) ERK signaling levels in response to phenomenon in panel (**b**). RAF inhibitor keeps ERK signaling within optimal zone.

Among the members of the Ras/RAF/MEK/ERK signaling cascades, RAF is a key direct effector of oncogenic Ras mutants and also a prominent target of oncogenic mutations. As the first kinase in this pathway, RAF has been thought as an ideal target for drug development against cancers. The first-generation RAF inhibitors, Vemurafenib [305,306], Dabrafenib [307], and Encorafenib [308], were developed and applied to treatment of BRAF(V600E)-harboring cancers as single agents or together with MEK inhibitors. These drugs achieved a promising efficacy at the initial therapeutic phase, although this efficacy was abrogated by quick-rising drug resistance. Mechanistic studies have shown that cancer cells reactivate this pathway upon drug treatment through two different ways: (1) upregulating the cellular level of active Ras, which leads to paradoxical activation of ERK signaling; and (2) alternative splicing of BRAF(V600E) to generate variants with truncated N-terminus, which enhances BRAF(V600E) homodimerization and decreases drug affinity. Interestingly, these drug-resistance cancer cells become addicted to RAF inhibitors, and drug withdrawal delays the growth of resistant cancers (Figure 5). This phenomenon can be explained by the concept of a “sweet-spot” for hyperactive Ras/RAF/MEK/ERK-signaling-driven cancer progression (Figure 5a). Specifically, cancer cells need optimal ERK signaling for growth, and ERK signaling that is too high will induce cell death or senescence and hence be toxic to cancer cells. Drug-resistant cancer cells have much higher ERK signaling than drug-sensitive cancer cells. Drug treatment decreases the ERK signaling in drug-resistance cancer cells to a level suitable for growth, while a drug withdrawal or “drug holiday” will inhibit their growth [309,310] (Figure 5b,c).

The paradoxical effect of the first-generation RAF inhibitors not only abrogated their efficacy but also induced secondary malignancies, a major side-effect of RAF inhibitors. To overcome the drug resistance of the first-generation RAF inhibitors, second-generation of RAF inhibitors were developed and underwent clinical trials; such inhibitors include pan-RAF inhibitors (such as LY3009120 [311], TAK632 [312], TAK580 [313], CCT3833 [314], BGB283 [262], BAL3833 [315], LXH254 [316], and RAF265 [317]), and paradox breakers (such as PLX8349 [318,319,320]). The pan-RAF inhibitors can inhibit both protomers in RAF dimers with similar affinity, while the paradox breakers induce an αC helix-out conformation upon loading and thereby prevent dimerization-driven transactivation.

To block hyperactive Ras/RAF/MEK/ERK signaling in cancers, MEK and ERK have also been used as targets for drug designs. Two MEK inhibitors (trametinib and cobimetinib) have been developed and approved for treating BRAF(V600E)-harboring cancers as single agents or together with RAF inhibitors, while ERK inhibitors are still undergoing clinical trials. In contrast to RAF inhibitors, these inhibitors have no paradoxical effect, but they do have a lower therapeutic index since they strongly inhibit this signaling pathway in normal cells [321,322]. This also implies that targeting downstream MEK/ERK may not be a good choice to treat Ras- or RAF-mutated cancers.

The development of RAF/MEK/ERK inhibitors has significantly improved targeted cancer therapy, and it has also accelerated our understanding of molecular mechanisms that tune the output of Ras signaling, which in turn facilitates the development of next-generation inhibitors. Studies on these inhibitors have revealed a dominant role of component interactions that include RAF/RAF, RAF/MEK, and MEK/MEK dimerizations in regulating Ras signaling. Since the structures of these complexes have been resolved, the development of allosteric inhibitors that break these interactions would be an attractive area of research in the coming years. In contrast to the first- and second-generation RAF inhibitors, these allosteric inhibitors should have many more advantages, such as no paradoxical effect and less off-target effects. 

## 12. Closing Remarks

The second-generation RAF inhibitors, which are expected to show longer-lasting effects on cancer growth, are now entering the clinical market [323]. Despite the enthusiasm, it is still not well understood how well cancers will respond to these drugs in the long term [262,324]. The studies on inhibitor-RAF interactions and the wild-type RAF response to the new inhibitors could help minimizing the off-target effects in patients. Determining the crystal structure and direct interaction between inhibitors/effectors would provide better insights into their mode of action in the pathways. This information not only could create potentially avenues for improved inhibition with better the drug specificity and efficacy; it could also reveal and validate novel allosteric targets within the pathway. Understanding the interactions between RAS/RAF/MEK mutants and their wild-type counterparts could result in the discovery of novel allosteric target sites to block the formation of dimers and tetramers between mutant and wild-type proteins. Besides the focus on RAS-downstream-signaling-partner studies, it is also interesting to follow the progress in the effort of targeting RAS functions, either directly or via modulating the functional post-translational prenylation pathway enzymes. With these comprehensive research efforts, we hope to have more specific and potent drugs targeting RAS/RAF/MEK/ERK signaling, to aid in the management of human cancers.

## References

[1] Rapp U.R., Todaro C. (1978). Generation of new mouse sarcoma viruses in cell culture. Science.

[2] Jansen H.W., Lurz R., Bister K., Bonner T.I., Mark G.E., Rapp U.R. (1984). Homologous cell-derived oncogenes in avian carcinoma virus MH2 and murine sarcoma virus 3611. Nature.

[3] Jansen H.W., Rückert B., Lurz R., Bister K. (1983). Two unrelated cell-derived sequences in the genome of avian leukemia and carcinoma inducing retrovirus MH2. EMBO J..

[4] Bonner T., O’Brien S., Nash W., Rapp U., Morton C., Leder P. (1984). The human homologs of the raf (mil) oncogene are located on human chromosomes 3 and 4. Science.

[5] Kozak C., Gunnell M.A., Rapp U.R. (1984). A new oncogene, c-raf, is located on mouse chromosome 6. J. Virol..

[6] Bonner T.I., Kerby S.B., Sutrave P., Gunnell M.A., Mark G., Rapp U.R. (1985). Structure and biological activity of human homologs of the raf/mil oncogene. Mol. Cell. Biol..

[7] Rapp U.R., Goldsborough M.D., Mark G.E., Bonner T.I., Groffen J., Reynolds F.H., Stephenson J.R. (1983). Structure and biological activity of v-raf, a unique oncogene transduced by a retrovirus. Proc. Natl. Acad. Sci. USA.

[8] Moelling K., Heimann B., Beimling P., Rapp U.R., Sander T. (1984). Serine- and threonine-specific protein kinase activities of purified gag–mil and gag–raf proteins. Nature.

[9] Kamata T., Feramisco J.R. (1984). Epidermal growth factor stimulates guanine nucleotide binding activity and phosphorylation of ras oncogene proteins. Nature.

[10] Mulcahy L.S., Smith M.R., Stacey D.W. (1985). Requirement for ras proto-oncogene function during serum-stimulated growth of NIH 3T3 cells. Nature.

[11] Smith M.R., DeGudicibus S.J., Stacey D.W. (1986). Requirement for c-ras proteins during viral oncogene transformation. Nature.

[12] Ambrosio L., Mahowald A.P., Perrimon N. (1989). Requirement of the Drosophila raf homologue for torso function. Nature.

[13] Han M., Golden A., Han Y., Sternberg P.W. (1993). C. elegans lin-45 raf gene participates in let-60 ras-stimulated vulval differentiation. Nature.

[14] Ahn N.G., Weiel J.E., Chan C.P., Krebs E.G. (1990). Identification of multiple epidermal growth factor-stimulated protein serine/threonine kinases from Swiss 3T3 cells. J. Biol. Chem..

[15] Ray L.B., Sturgill T.W. (1988). Characterization of insulin-stimulated microtubule-associated protein kinase. J. Biol. Chem..

[16] Rossomando A.J., Payne D.M., Weber M.J., Sturgill T.W. (1989). Evidence that pp42, a major tyrosine kinase target protein, is a mitogen-activated serine/threonine protein kinase. Proc. Natl. Acad. Sci. USA.

[17] Boulton T.G., Nye S.H., Robbins D.J., Ip N.Y., Radzlejewska E., Morgenbesser S.D., Depinho R.A., Panayotatos N., Cobb M.H., Yancopoulos G.D. (1991). ERKs: A family of protein-serine/threonine kinases that are activated and tyrosine phosphorylated in response to insulin and NGF. Cell.

[18] Crews C.M., Erikson R.L. (1992). Purification of a murine protein-tyrosine/threonine kinase that phosphorylates and activates the Erk-1 gene product: Relationship to the fission yeast byr1 gene product. Proc. Natl. Acad. Sci. USA.

[19] Kyriakis J.M., App H., Zhang X., Banerjee P., Brautigan D.L., Rapp U.R., Avruch J. (1992). Raf-1 activates MAP kinase-kinase. Nature.

[20] Alessi D.R., Saito Y., Campbell D.G., Cohen P., Sithanandam G., Rapp U., Ashworth A., Marshall C.J., Cowley S. (1994). Identification of the sites in MAP kinase kinase-1 phosphorylated by p74raf-1. EMBO J..

[21] Kolch W., Heidecker G., Lloyd P., Rapp U.R. (1991). Raf-1 protein kinase is required for growth of induced NIH/3T3 cells. Nature.

[22] Zhang X.-F., Settleman J., Kyriakis J., Takeuchi-Suzuki E., Elledge S.J., Marshall M.S., Bruder J.T., Rapp U.R., Avruch J. (1993). Normal and oncogenic p21ras proteins bind to the amino-terminal regulatory domain of c-Raf-1. Nature.

[23] Van Aelst L., Barr M., Marcus S., Polverino A., Wigler M. (1993). Complex formation between RAS and RAF and other protein kinases. Proc. Natl. Acad. Sci. USA.

[24] Vojtek A.B., Hollenberg S.M., Cooper J.A. (1993). Mammalian Ras interacts directly with the serine/threonine kinase raf. Cell.

[25] Moodie S.A., Willumsen B.M., Weber M.J., Wolfman A. (1993). Complexes of Ras.GTP with Raf-1 and mitogen-activated protein kinase kinase. Science.

[26] Davies H., Bignell G.R., Cox C., Stephens P., Edkins S., Clegg S., Teague J., Woffendin H., Garnett M.J., Bottomley W. (2002). Mutations of the BRAF gene in human cancer. Nature.

[27] Garnett M.J., Rana S., Paterson H., Barford D., Marais R. (2005). Wild-Type and Mutant B-RAF Activate C-RAF through Distinct Mechanisms Involving Heterodimerization. Mol. Cell.

[28] Winter-Vann A.M., Casey P.J. (2005). Post-prenylation-processing enzymes as new targets in oncogenesis. Nat. Rev. Cancer.

[29] Jang H., Abraham S.J., Chavan T.S., Hitchinson B., Khavrutskii L., Tarasova N.I., Nussinov R., Gaponenko V. (2015). Mechanisms of Membrane Binding of Small GTPase K-Ras4B Farnesylated Hypervariable Region. J. Biol. Chem..

[30] Hobbs G.A., Der C.J. (2019). RAS Mutations Are Not Created Equal. Cancer Discov..

[31] Li S., Balmain A., Counter C.M. (2018). A model for RAS mutation patterns in cancers: Finding the sweet spot. Nat. Rev. Cancer.

[32] Cox A.D., Der C.J. (2010). Ras history. Small GTPases.

[33] Castellano E., Santos E. (2011). Functional specificity of ras isoforms: So similar but so different. Genes Cancer.

[34] Hancock J.F. (2003). Ras proteins: Different signals from different locations. Nat. Rev. Mol. Cell Biol..

[35] Murugan A.K., Grieco M., Tsuchida N. (2019). RAS mutations in human cancers: Roles in precision medicine. Semin. Cancer Biol..

[36] Yan J., Roy S., Apolloni A., Lane A., Hancock J.F. (1998). Ras Isoforms Vary in Their Ability to Activate Raf-1 and Phosphoinositide 3-Kinase. J. Biol. Chem..

[37] Millán O., Ballester A., Castrillo A., Oliva J.L. de la, Través P.G., Rojas J.M., Boscá L. (2003). H-Ras-specific activation of NF-κB protects NIH 3T3 cells against stimulus-dependent apoptosis. Oncogene.

[38] Hobbs G.A., Der C.J., Rossman K.L. (2016). RAS isoforms and mutations in cancer at a glance. J. Cell Sci..

[39] McCormick F. (1989). ras GTPase activating protein: Signal transmitter and signal terminator. Cell.

[40] Hennig A., Markwart R., Esparza-Franco M.A., Ladds G., Rubio I. (2015). Ras activation revisited: Role of GEF and GAP systems. Biol. Chem..

[41] Aoki Y., Niihori T., Inoue S., Matsubara Y. (2015). Recent advances in RASopathies. J. Hum. Genet..

[42] Quilliam L.A., Castro A.F., Rogers-Graham K.S., Martin C.B., Der C.J., Bi C. (1999). M-Ras/R-Ras3, a transforming ras protein regulated by Sos1, GRF1, and p120 Ras GTPase-activating protein, interacts with the putative Ras effector AF6. J. Biol. Chem..

[43] Maertens O., Johnson B., Hollstein P., Frederick D.T., Cooper Z.A., Messiaen L., Bronson R.T., McMahon M., Granter S., Flaherty K. (2013). Elucidating distinct roles for NF1 in melanomagenesis. Cancer Discov..

[44] Casey P.J., Solski P.A., Der C.J., Buss J.E. (1989). p21ras is modified by a farnesyl isoprenoid. Proc. Natl. Acad. Sci. USA.

[45] Sebti S.M. (2005). Protein farnesylation: Implications for normal physiology, malignant transformation, and cancer therapy. Cancer Cell.

[46] Wang M., Casey P.J. (2016). Protein prenylation: Unique fats make their mark on biology. Nat. Rev. Mol. Cell Biol..

[47] Williams C. (2013). A new signaling paradigm to control the prenylation and trafficking of small GTPases. Cell Cycle.

[48] Zhang F.L., Casey P.J. (1996). Protein prenylation: Molecular Mechanisms and Functional Consequences. Annu. Rev. Biochem..

[49] Lerner E.C., Qian Y., Blaskovich M.A., Fossum R.D., Vogt A., Sun J., Cox A.D., Der C.J., Hamilton A.D., Sebti S.M. (1995). Ras CAAX peptidomimetic FTI-277 selectively blocks oncogenic Ras signaling by inducing cytoplasmic accumulation of inactive Ras-Raf complexes. J. Biol. Chem..

[50] Casey P.J., Thissen J.A., Moomaw J.F. (1991). Enzymatic modification of proteins with a geranylgeranyl isoprenoid. Proc. Natl. Acad. Sci. USA.

[51] Hancock J.F., Magee A.I., Childs J.E., Marshall C.J. (1989). All ras proteins are polyisoprenylated but only some are palmitoylated. Cell.

[52] Buss J.E., Quilliam L.A., Kato K., Casey P.J., Solski P.A., Wong G., Clark R., McCormick F., Bokoch G.M., Der C.J. (1991). The COOH-terminal domain of the Rap1A (Krev-1) protein is isoprenylated and supports transformation by an H-Ras:Rap1A chimeric protein. Mol. Cell. Biol..

[53] Wright L.P., Philips M.R. (2006). Thematic review series: Lipid Posttranslational Modifications CAAX modification and membrane targeting of Ras. J. Lipid Res..

[54] Boyartchuk V.L. (1997). Modulation of Ras and a-Factor Function by Carboxyl-Terminal Proteolysis. Science.

[55] Bergo M.O., Gavino B.J., Hong C., Beigneux A.P., McMahon M., Casey P.J., Young S.G. (2004). Inactivation of Icmt inhibits transformation by oncogenic K-Ras and B-Raf. J. Clin. Investig..

[56] Bergo M.O., Leung G.K., Ambroziak P., Otto J.C., Casey P.J., Young S.G. (2000). Targeted Inactivation of the Isoprenylcysteine Carboxyl Methyltransferase Gene Causes Mislocalization of K-Ras in Mammalian Cells. J. Biol. Chem..

[57] Winter-Vann A.M., Kamen B.A., Bergo M.O., Young S.G., Melnyk S., James S.J., Casey P.J. (2003). Targeting Ras signaling through inhibition of carboxyl methylation: An unexpected property of methotrexate. Proc. Natl. Acad. Sci. USA.

[58] Inouye K., Mizutani S., Koide H., Kaziro Y. (2000). Formation of the Ras Dimer Is Essential for Raf-1 Activation. J. Biol. Chem..

[59] Varma R., Mayor S. (1998). GPI-anchored proteins are organized in submicron domains at the cell surface. Nature.

[60] Prior I.A., Muncke C., Parton R.G., Hancock J.F. (2003). Direct visualization of Ras proteins in spatially distinct cell surface microdomains. J. Cell Biol..

[61] Plowman S.J., Muncke C., Parton R.G., Hancock J.F. (2005). H-ras, K-ras, and inner plasma membrane raft proteins operate in nanoclusters with differential dependence on the actin cytoskeleton. Proc. Natl. Acad. Sci. USA.

[62] Zhou Y., Hancock J.F. (2015). Ras nanoclusters: Versatile lipid-based signaling platforms. Biochim. Biophys. Acta Mol. Cell Res..

[63] Tian T., Harding A., Inder K., Plowman S., Parton R.G., Hancock J.F. (2007). Plasma membrane nanoswitches generate high-fidelity Ras signal transduction. Nat. Cell Biol..

[64] Huleihel M., Goldsborough M., Cleveland J., Gunnell M., Bonner T., Rapp U.R. (1986). Characterization of murine A-raf, a new oncogene related to the v-raf oncogene. Mol. Cell. Biol..

[65] Beck T.W., Huleihel M., Gunnell M., Bonner T.I., Rapp U.R. (1987). The complete coding sequence of the human A-raf-1 oncogene and transforming activity of a human A-raf carrying retrovirus. Nucl. Acids Res..

[66] Ikawa S., Fukui M., Ueyama Y., Tamaoki N., Yamamoto T., Toyoshima K. (1988). B-raf, a new member of the raf family, is activated by DNA rearrangement. Mol. Cell. Biol..

[67] Daum G., Eisenmann-Tappe I., Fries H.-W., Troppmair J., Rapp U.R. (1994). The ins and outs of Raf kinases. Trends Biochem. Sci..

[68] Wellbrock C., Karasarides M., Marais R. (2004). The RAF proteins take centre stage. Nat. Rev. Mol. Cell Biol..

[69] Nassar N., Horn G., Herrmann C.A., Scherer A., McCormick F., Wittinghofer A. (1995). The 2.2 Å crystal structure of the Ras-binding domain of the serine/threonine kinase c-Raf1 in complex with RaplA and a GTP analogue. Nature.

[70] Scheffler J.E., Waugh D.S., Bekesi E., Kiefer S.E., LoSardo J.E., Neri A., Prinzo K.M., Tsao K.L., Wegrzynski B., Emerson S.D. (1994). Characterization of a 78-residue fragment of c-Raf-1 that comprises a minimal binding domain for the interaction with Ras-GTP. J. Biol. Chem..

[71] Chuang E., Barnard D., Hettich L., Zhang X.F., Avruch J., Marshall M.S. (1994). Critical binding and regulatory interactions between Ras and Raf occur through a small, stable N-terminal domain of Raf and specific Ras effector residues. Mol. Cell. Biol..

[72] Emerson S.D., Waugh D.S., Scheffler J.E., Tsao K.-L., Prinzo K.M., Fry D.C. (1994). Chemical Shift Assignments and Folding Topology of the RAS-Binding Domain of Human RAF-1 As Determined by Heteronuclear Three-Dimensional NMR Spectroscopy. Biochemistry.

[73] Mott H.R., Carpenter J.W., Zhong S., Ghosh S., Bell R.M., Campbell S.L. (1996). The solution structure of the Raf-1 cysteine-rich domain: A novel ras and phospholipid binding site. Proc. Natl. Acad. Sci. USA.

[74] Diaz B., Barnard D., Filson A., MacDonald S., King A., Marshall M. (1997). Phosphorylation of Raf-1 serine 338-serine 339 is an essential regulatory event for Ras-dependent activation and biological signaling. Mol. Cell. Biol..

[75] Mason C.S., Springer C.J., Cooper R.G., Superti-Furga G., Marshall C.J., Marais R. (1999). Serine and tyrosine phosphorylations cooperate in Raf-1, but not B-Raf activation. EMBO J..

[76] Dhillon A.S., Yip Y.Y., Grindlay G.J., Pakay J.L., Dangers M., Hillmann M., Clark W., Pitt A., Mischak H., Kolch W. (2009). The C-terminus of Raf-1 acts as a 14-3-3-dependent activation switch. Cell. Signal..

[77] Kondo Y., Ognjenović J., Banerjee S., Karandur D., Merk A., Kulhanek K., Wong K., Roose J.P., Subramaniam S., Kuriyan J. (2019). Cryo-EM structure of a dimeric B-Raf:14-3-3 complex reveals asymmetry in the active sites of B-Raf kinases. Science.

[78] Muslin A.J., Tanner J.W., Allen P.M., Shaw A.S. (1996). Interaction of 14-3-3 with Signaling Proteins Is Mediated by the Recognition of Phosphoserine. Cell.

[79] Morrison D.K., Heidecker G., Rapp U.R., Copeland T.D., Morrisonst D.K., Heideckerq G., Rappq U.R., Copeland T.D. (1993). Identification of the major phosphorylation sites of the Raf-1 kinase. J. Biol. Chem..

[80] Hu J., Stites E.C., Yu H., Germino E.A., Meharena H.S., Stork P.J.S., Kornev A.P., Taylor S.S., Shaw A.S. (2013). Allosteric Activation of Functionally Asymmetric RAF Kinase Dimers. Cell.

[81] Blasco R.B., Francoz S., Santamaría D., Cañamero M., Dubus P., Charron J., Baccarini M., Barbacid M. (2011). c-Raf, but Not B-Raf, Is Essential for Development of K-Ras Oncogene-Driven Non-Small Cell Lung Carcinoma. Cancer Cell.

[82] Karreth F.A., Frese K.K., DeNicola G.M., Baccarini M., Tuveson D.A. (2011). C-Raf is required for the initiation of lung cancer by K-Ras(G12D). Cancer Discov..

[83] Lito P., Saborowski A., Yue J., Solomon M., Joseph E., Gadal S., Saborowski M., Kastenhuber E., Fellmann C., Ohara K. (2014). Disruption of CRAF-Mediated MEK Activation Is Required for Effective MEK Inhibition in KRAS Mutant Tumors. Cancer Cell.

[84] Yuan J., Ng W.H., Lam P.Y.P.P., Wang Y., Xia H., Yap J., Guan S.P., Lee A.S.G.G., Wang M., Baccarini M. (2018). The dimer-dependent catalytic activity of RAF family kinases is revealed through characterizing their oncogenic mutants. Oncogene.

[85] Yap J., Yuan J., Tee Z.H., Huang X., Ng W.H., Hu J. (2019). Characterize Disease-related Mutants of RAF Family Kinases by Using a Set of Practical and Feasible Methods. JoVE.

[86] Haling J.R., Sudhamsu J., Yen I., Sideris S., Sandoval W., Phung W., Bravo B.J., Giannetti A.M., Peck A., Masselot A. (2014). Structure of the BRAF-MEK Complex Reveals a Kinase Activity Independent Role for BRAF in MAPK Signaling. Cancer Cell.

[87] Kornev A.P., Haste N.M., Taylor S.S., Ten Eyck L.F. (2006). Surface comparison of active and inactive protein kinases identifies a conserved activation mechanism. Proc. Natl. Acad. Sci. USA.

[88] Taylor S.S., Kornev A.P. (2011). Protein kinases: Evolution of dynamic regulatory proteins. Trends Biochem. Sci..

[89] Taylor S.S., Shaw A., Hu J., Meharena H.S., Kornev A. (2013). Pseudokinases from a structural perspective. Biochem. Soc. Trans..

[90] Shaw A.S., Kornev A.P., Hu J., Ahuja L.G., Taylor S.S. (2014). Kinases and Pseudokinases: Lessons from RAF. Mol. Cell. Biol..

[91] Hu J., Ahuja L.G., Meharena H.S., Kannan N., Kornev A.P., Taylor S.S., Shaw A.S. (2015). Kinase Regulation by Hydrophobic Spine Assembly in Cancer. Mol. Cell. Biol..

[92] Kim J., Ahuja L.G., Chao F., Xia Y., Mcclendon C.L., Kornev A.P., Taylor S.S., Veglia G. (2017). A dynamic hydrophobic core orchestrates allostery in protein kinases. Sci. Adv..

[93] Vijayan R.S.K., He P., Modi V., Duong-Ly K.C., Ma H., Peterson J.R., Dunbrack R.L., Levy R.M. (2015). Conformational analysis of the DFG-out kinase motif and biochemical profiling of structurally validated type II inhibitors. J. Med. Chem..

[94] Treiber D.K., Shah N.P. (2013). Ins and Outs of Kinase DFG Motifs. Chem. Biol..

[95] Thevakumaran N., Lavoie H., Critton D.A., Tebben A., Marinier A., Sicheri F., Therrien M. (2015). Crystal structure of a BRAF kinase domain monomer explains basis for allosteric regulation. Nat. Struct. Mol. Biol..

[96] Terrell E.M., Morrison D.K. (2019). Ras-Mediated Activation of the Raf Family Kinases. Cold Spring Harb. Perspect. Med..

[97] Wojnowski L., Stancato L.F., Zimmer A.M., Hahn H., Beck T.W., Larner A.C., Rapp U.R., Zimmer A. (1998). Craf-1 protein kinase is essential for mouse development. Mech. Dev..

[98] Mikula M., Schreiber M., Husak Z., Kucerova L., Rüth J., Wieser R., Zatloukal K., Beug H., Wagner E.F., Baccarini M. (2001). Embryonic lethality and fetal liver apoptosis in mice lacking the c-raf-1 gene. EMBO J..

[99] Wojnowski L., Zimmer A.M., Beck T.W., Hahn H., Bernal R., Rapp U.R., Zimmer A. (1997). Endothelial apoptosis in Braf-deficient mice. Nat. Genet..

[100] Wiese S., Pei G., Karch C., Troppmair J., Holtmann B., Rapp U.R., Sendtner M. (2001). Specific function of B-Raf in mediating survival of embryonic motoneurons and sensory neurons. Nat. Neurosci..

[101] Pritchard C.A., Bolin L., Slattery R., Murray R., McMahon M. (1996). Post-natal lethality and neurological and gastrointestinal defects in mice with targeted disruption of the A-Raf protein kinase gene. Curr. Biol..

[102] Hüser M., Luckett J., Chiloeches A., Mercer K., Iwobi M., Giblett S., Sun X.M., Brown J., Marais R., Pritchard C. (2001). MEK kinase activity is not necessary for Raf-1 function. EMBO J..

[103] Piazzolla D., Meissl K., Kucerova L., Rubiolo C., Baccarini M. (2005). Raf-1 sets the threshold of Fas sensitivity by modulating Rok-alpha signaling. J. Cell Biol..

[104] Ehrenreiter K., Piazzolla D., Velamoor V., Sobczak I., Small J.V., Takeda J., Leung T., Baccarini M. (2005). Raf-1 regulates Rho signaling and cell migration. J. Cell Biol..

[105] Niault T., Sobczak I., Meissl K., Weitsman G., Piazzolla D., Maurer G., Kern F., Ehrenreiter K., Hamerl M., Moarefi I. (2009). From autoinhibition to inhibition in trans: The Raf-1 regulatory domain inhibits Rok-alpha kinase activity. J. Cell Biol..

[106] Ehrenreiter K., Kern F., Velamoor V., Meissl K., Galabova-Kovacs G., Sibilia M., Baccarini M. (2009). Raf-1 Addiction in Ras-Induced Skin Carcinogenesis. Cancer Cell.

[107] Sanclemente M., Francoz S., Esteban-Burgos L., Bousquet-Mur E., Djurec M., Lopez-Casas P.P., Hidalgo M., Guerra C., Drosten M., Musteanu M. (2018). c-RAF Ablation Induces Regression of Advanced Kras/Trp53 Mutant Lung Adenocarcinomas by a Mechanism Independent of MAPK Signaling. Cancer Cell.

[108] Rubiolo C., Piazzolla D., Meissl K., Beug H., Huber J.C., Kolbus A., Baccarini M. (2006). A balance between Raf-1 and Fas expression sets the pace of erythroid differentiation. Blood.

[109] Kolbus A., Pilat S., Husak Z., Deiner E.M., Stengl G., Beug H., Baccarini M. (2002). Raf-1 Antagonizes Erythroid Differentiation by Restraining Caspase Activation. J. Exp. Med..

[110] Wang H.-G., Rapp U.R., Reed J.C. (1996). Bcl-2 Targets the Protein Kinase Raf-1 to Mitochondria. Cell.

[111] Panka D.J., Atkins M.B., Mier J.W. (2006). Targeting the Mitogen-Activated Protein Kinase Pathway in the Treatment of Malignant Melanoma. Clin. Cancer Res..

[112] Baumann B., Weber C.K., Troppmair J., Whiteside S., Israel A., Rapp U.R., Wirth T. (2000). Raf induces NF-kappaB by membrane shuttle kinase MEKK1, a signaling pathway critical for transformation. Proc. Natl. Acad. Sci. USA.

[113] Troppmair J., Hartkamp J., Rapp U.R. (1998). Activation of NF-κB by oncogenic Raf in HEK 293 cells occurs through autocrine recruitment of the stress kinase cascade. Oncogene.

[114] Norris J.L., Baldwin A.S. (1999). Oncogenic Ras Enhances NF-κB Transcriptional Activity through Raf-dependent and Raf-independent Mitogen-activated Protein Kinase Signaling Pathways. J. Biol. Chem..

[115] Majewski M., Nieborowska-Skorska M., Salomoni P., Slupianek A., Reiss K., Trotta R., Calabretta B., Skorski T. (1999). Activation of Mitochondrial Raf-1 Is Involved in the Antiapoptotic Effects of Akt. Cancer Res..

[116] Wu X., Carr H.S., Dan I., Ruvolo P.P., Frost J.A. (2008). p21 activated kinase 5 activates Raf-1 and targets it to mitochondria. J. Cell. Biochem..

[117] Jin S., Zhuo Y., Guo W., Field J. (2005). p21-activated Kinase 1 (Pak1)-dependent Phosphorylation of Raf-1 Regulates Its Mitochondrial Localization, Phosphorylation of BAD, and Bcl-2 Association. J. Biol. Chem..

[118] Hindley A., Kolch W. (2007). Raf-1 and B-Raf promote protein kinase C theta interaction with BAD. Cell. Signal..

[119] Le Mellay V., Troppmair J., Benz R., Rapp U.R. (2002). Negative regulation of mitochondrial VDAC channels by C-Raf kinase. BMC Cell Biol..

[120] Chen J., Fujii K., Zhang L., Roberts T., Fu H. (2001). Raf-1 promotes cell survival by antagonizing apoptosis signal-regulating kinase 1 through a MEK-ERK independent mechanism. Proc. Natl. Acad. Sci. USA.

[121] Alavi A.S., Acevedo L., Min W., Cheresh D.A. (2007). Chemoresistance of Endothelial Cells Induced by Basic Fibroblast Growth Factor Depends on Raf-1–Mediated Inhibition of the Proapoptotic Kinase, ASK1. Cancer Res..

[122] Tobiume K., Matsuzawa A., Takahashi T., Nishitoh H., Morita K., Takeda K., Minowa O., Miyazono K., Noda T., Ichijo H. (2001). ASK1 is required for sustained activations of JNK/p38 MAP kinases and apoptosis. EMBO Rep..

[123] O’Neill E., Rushworth L., Baccarini M., Kolch W. (2004). Role of the kinase MST2 in suppression of apoptosis by the proto-oncogene product Raf-1. Science.

[124] Yamaguchi O., Watanabe T., Nishida K., Kashiwase K., Higuchi Y., Takeda T., Hikoso S., Hirotani S., Asahi M., Taniike M. (2004). Cardiac-specific disruption of the c-raf-1 gene induces cardiac dysfunction and apoptosis. J. Clin. Investig..

[125] Rauch J., Kolch W. (2019). Spatial regulation of ARAF controls the MST2-Hippo pathway. Small GTPases.

[126] Rauch J., O’Neill E., Mack B., Matthias C., Munz M., Kolch W., Gires O. (2010). Heterogeneous nuclear ribonucleoprotein H blocks MST2-mediated apoptosis in cancer cells by regulating A-Raf transcription. Cancer Res..

[127] Christofk H.R., Vander Heiden M.G., Harris M.H., Ramanathan A., Gerszten R.E., Wei R., Fleming M.D., Schreiber S.L., Cantley L.C. (2008). The M2 splice isoform of pyruvate kinase is important for cancer metabolism and tumour growth. Nature.

[128] Le Mellay V., Houben R., Troppmair J., Hagemann C., Mazurek S., Frey U., Beigel J., Weber C., Benz R., Eigenbrodt E. (2002). Regulation of glycolysis by Raf protein serine/threonine kinases. Adv. Enzyme Regul..

[129] Mercer K., Giblett S., Oakden A., Brown J., Marais R., Pritchard C. (2005). A-Raf and Raf-1 work together to influence transient ERK phosphorylation and Gl/S cell cycle progression. Oncogene.

[130] Wojnowski L., Stancato L.F., Larner A.C., Rapp U.R., Zimmer A. (2000). Overlapping and specific functions of Braf and Craf-1 proto-oncogenes during mouse embryogenesis. Mech. Dev..

[131] Mercer K., Giblett S., Green S., Lloyd D., DaRocha Dias S., Plumb M., Marais R., Pritchard C. (2005). Expression of endogenous oncogenic V600EB-raf induces proliferation and developmental defects in mice and transformation of primary fibroblasts. Cancer Res..

[132] Cutler R.E., Stephens R.M., Saracino M.R., Morrison D.K. (1998). Autoregulation of the Raf-1 serine/threonine kinase. Proc. Natl. Acad. Sci. USA.

[133] Morrison D.K., Cutler R.E. (1997). The complexity of Raf-1 regulation. Curr. Opin. Cell Biol..

[134] Jin T., Lavoie H., Sahmi M., David M., Hilt C., Hammell A., Therrien M. (2017). RAF inhibitors promote RAS-RAF interaction by allosterically disrupting RAF autoinhibition. Nat. Commun..

[135] Hey F., Pritchard C. (2013). A New Mode of RAF Autoregulation: A Further Complication in the Inhibitor Paradox. Cancer Cell.

[136] Sanchez-Vega F., Mina M., Armenia J., Chatila W.K., Luna A., La K.C., Dimitriadoy S., Liu D.L., Kantheti H.S., Saghafinia S. (2018). Oncogenic Signaling Pathways in The Cancer Genome Atlas. Cell.

[137] Ahearn I.M., Haigis K., Bar-Sagi D., Philips M.R. (2011). Regulating the regulator: Post-translational modification of RAS. Nat. Rev. Mol. Cell Biol..

[138] Clark G.J., Drugan J.K., Terrell R.S., Bradham C., Der C.J., Bell R.M., Campbell S. (1996). Peptides containing a consensus Ras binding sequence from Raf-1 and theGTPase activating protein NF1 inhibit Ras function. Proc. Natl. Acad. Sci. USA.

[139] Pacold M.E., Suire S., Perisic O., Lara-Gonzalez S., Davis C.T., Walker E.H., Hawkins P.T., Stephens L., Eccleston J.F., Williams R.L. (2000). Crystal Structure and Functional Analysis of Ras Binding to Its Effector Phosphoinositide 3-Kinase γ. Cell.

[140] Fabian J.R., Vojtek A.B., Cooper J.A., Morrison D.K. (1994). A single amino acid change in Raf-1 inhibits Ras binding and alters Raf-1 function. Proc. Natl. Acad. Sci. USA.

[141] Im E., von Lintig F.C., Chen J., Zhuang S., Qui W., Chowdhury S., Worley P.F., Boss G.R., Pilz R.B. (2002). Rheb is in a high activation state and inhibits B-Raf kinase in mammalian cells. Oncogene.

[142] Vossler M.R., Yao H., York R.D., Pan M.-G., Rim C.S., Stork P.J.S. (1997). cAMP Activates MAP Kinase and Elk-1 through a B-Raf- and Rap1-Dependent Pathway. Cell.

[143] Fischer A., Hekman M., Kuhlmann J., Rubio I., Wiese S., Rapp U.R. (2007). B- and C-RAF Display Essential Differences in Their Binding to Ras: The isotype-specific n terminus of b-RAF facilitates ras binding. J. Biol. Chem..

[144] Luo Z., Diaz B., Marshall M.S., Avruch J. (1997). An intact Raf zinc finger is required for optimal binding to processed Ras and for ras-dependent Raf activation in situ. Mol. Cell. Biol..

[145] Williams J.G., Drugan J.K., Yi G.S., Clark G.J., Der C.J., Campbell S.L. (2000). Elucidation of binding determinants and functional consequences of Ras/Raf-cysteine-rich domain interactions. J. Biol. Chem..

[146] Winkler D.G., Cutler R.E., Drugan J.K., Campbell S., Morrison D.K., Cooper J.A. (1998). Identification of Residues in the Cysteine-rich Domain of Raf-1 That Control Ras Binding and Raf-1 Activity. J. Biol. Chem..

[147] Thapar R., Williams J.G., Campbell S.L. (2004). NMR Characterization of Full-length Farnesylated and Non-farnesylated H-Ras and its Implications for Raf Activation. J. Mol. Biol..

[148] Ding J., Tchaicheeyan O., Ambrosio L. (2010). Drosophila Raf’s N Terminus Contains a Novel Conserved Region and Can Contribute to Torso RTK Signaling. Genetics.

[149] Kubicek M., Pacher M., Abraham D., Podar K., Eulitz M., Baccarini M. (2002). Dephosphorylation of Ser-259 regulates Raf-1 membrane association. J. Biol. Chem..

[150] Tran N.H., Wu X., Frost J.A. (2005). B-Raf and Raf-1 Are Regulated by Distinct Autoregulatory Mechanisms. J. Biol. Chem..

[151] Abraham D., Podar K., Pacher M., Kubicek M., Welzel N., Hemmings B.A., Dilworth S.M., Mischak H., Kolch W., Baccarini M. (2000). Raf-1-associated protein phosphatase 2A as a positive regulator of kinase activation. J. Biol. Chem..

[152] Jaumot M., Hancock J.F. (2001). Protein phosphatases 1 and 2A promote Raf-1 activation by regulating 14-3-3 interactions. Oncogene.

[153] Ory S., Zhou M., Conrads T.P., Veenstra T.D., Morrison D.K. (2003). Protein Phosphatase 2A Positively Regulates Ras Signaling by Dephosphorylating KSR1 and Raf-1 on Critical 14-3-3 Binding Sites. Curr. Biol..

[154] Eisenhardt A.E., Sprenger A., Röring M., Herr R., Weinberg F., Köhler M., Braun S., Orth J., Diedrich B., Lanner U. (2016). Phospho-proteomic analyses of B-Raf protein complexes reveal new regulatory principles. Oncotarget.

[155] Luo Z., Tzivion G., Belshaw P.J., Vavvas D., Marshall M., Avruch J. (1996). Oligomerization activates c-Raf-1 through a Ras-dependent mechanism. Nature.

[156] Farrar M.A., Alberola-lla J., Perlmutter R.M. (1996). Activation of the Raf-1 kinase cascade by coumermycin-induced dimerization. Nature.

[157] Weber C.K., Slupsky J.R., Kalmes H.A., Rapp U.R. (2001). Active Ras Induces Heterodimerization of cRaf and BRaf 1. Cancer Res..

[158] Rajakulendran T., Sahmi M., Lefrançois M., Sicheri F., Therrien M. (2009). A dimerization-dependent mechanism drives RAF catalytic activation. Nature.

[159] Rushworth L.K., Hindley A.D., O’Neill E., Kolch W. (2006). Regulation and role of Raf-1/B-Raf heterodimerization. Mol. Cell. Biol..

[160] Heidorn S.J., Milagre C., Whittaker S., Nourry A., Niculescu-Duvas I., Dhomen N., Hussain J., Reis-Filho J.S., Springer C.J., Pritchard C. (2010). Kinase-Dead BRAF and Oncogenic RAS Cooperate to Drive Tumor Progression through CRAF. Cell.

[161] Kwong L.N., Chin L. (2010). The Brothers RAF. Cell.

[162] Hu J., Yu H., Kornev A.P., Zhao J., Filbert E.L., Taylor S.S., Shaw A.S. (2011). Mutation that blocks ATP binding creates a pseudokinase stabilizing the scaffolding function of kinase suppressor of Ras, CRAF and BRAF. Proc. Natl. Acad. Sci. USA.

[163] Baljuls A., Mahr R., Schwarzenau I., Müller T., Polzien L., Hekman M., Rapp U.R. (2011). Single Substitution within the RKTR Motif Impairs Kinase Activity but Promotes Dimerization of RAF Kinase. J. Biol. Chem..

[164] Jambrina P.G., Rauch N., Pilkington R., Rybakova K., Nguyen L.K., Kholodenko B.N., Buchete N.-V., Kolch W., Rosta E. (2016). Phosphorylation of RAF Kinase Dimers Drives Conformational Changes that Facilitate Transactivation. Angew. Chem. Int. Ed..

[165] Poulikakos P.I., Persaud Y., Janakiraman M., Kong X., Ng C., Moriceau G., Shi H., Atefi M., Titz B., Gabay M.T. (2011). RAF inhibitor resistance is mediated by dimerization of aberrantly spliced BRAF(V600E). Nature.

[166] Dunham I., Kundaje A., Aldred S.F., Collins P.J., Davis C.A., Doyle F., Epstein C.B., Frietze S., Harrow J., Kaul R. (2012). An integrated encyclopedia of DNA elements in the human genome. Nature.

[167] Palanisamy N., Ateeq B., Kalyana-Sundaram S., Pflueger D., Ramnarayanan K., Shankar S., Han B., Cao Q., Cao X., Suleman K. (2010). Rearrangements of the RAF kinase pathway in prostate cancer, gastric cancer and melanoma. Nat. Med..

[168] Jain P., Fierst T.M., Han H.J., Smith T.E., Vakil A., Storm P.B., Resnick A.C., Waanders A.J. (2017). CRAF gene fusions in pediatric low-grade gliomas define a distinct drug response based on dimerization profiles. Oncogene.

[169] Stransky N., Cerami E., Schalm S., Kim J.L., Lengauer C. (2014). The landscape of kinase fusions in cancer. Nat. Commun..

[170] Tran N.H., Frost J.A. (2003). Phosphorylation of Raf-1 by p21-activated Kinase 1 and Src Regulates Raf-1 Autoinhibition. J. Biol. Chem..

[171] Williams N.G., Roberts T.M., Li P. (1992). Both p21ras and pp60v-src are required, but neither alone is sufficient, to activate the Raf-1 kinase. Proc. Natl. Acad. Sci. USA.

[172] Thompson P.A., Ledbetter J.A., Rapp U.R., Bolen J.B. (1991). The Raf-1 serine-threonine kinase is a substrate for the p56lck protein tyrosine kinase in human T-cells. Cell Growth Differ..

[173] Fabian J.R., Daar I.R.A., Morrison D.K. (1993). Critical Tyrosine Residues Regulate the Enzymatic and Biological Activity of Raf-1 Kinase. Mol. Cell. Biol..

[174] Cleghon V., Morrison D.K. (1994). Raf-1 Interacts with Fyn and Src in a Non-phosphotyrosine-dependent Manner. J. Biol. Chem..

[175] Marais R., Light Y., Paterson H.F., Marshall C.J. (1995). Ras recruits Raf-1 to the plasma membrane for activation by tyrosine phosphorylation. EMBO J..

[176] Chiloeches A., Mason C.S., Marais R. (2001). S338 phosphorylation of Raf-1 is independent of phosphatidylinositol 3-kinase and Pak3. Mol. Cell. Biol..

[177] Chaudhary A., King W.G., Mattaliano M.D., Frost J.A., Diaz B., Morrison D.K., Cobb M.H., Marshall M.S., Brugge J.S. (2000). Phosphatidylinositol 3-kinase regulates Raf1 through Pak phosphorylation of serine 338. Curr. Biol..

[178] King A.J., Sun H., Diaz B., Barnard D., Miao W., Bagrodia S., Marshall M.S. (1998). The protein kinase Pak3 positively regulates Raf-1 activity through phosphorylation of serine 338. Nature.

[179] Xia K., Mukhopadhyay N.K., Inhorn R.C., Barber D.L., Rose P.E., Lee R.S., Narsimhan R.P., D’Andrea A.D., Griffin J.D., Roberts T.M. (1996). The cytokine-activated tyrosine kinase JAK2 activates Raf-1 in a p21ras-dependent manner. Proc. Natl. Acad. Sci. USA.

[180] Ritt D.A., Zhou M., Conrads T.P., Veenstra T.D., Copeland T.D., Morrison D.K. (2007). CK2 Is a Component of the KSR1 Scaffold Complex that Contributes to Raf Kinase Activation. Curr. Biol..

[181] Salzano M., Rusciano M.R., Russo E., Bifulco M., Postiglione L., Vitale M. (2012). Calcium/calmodulin-dependent protein kinase II (CaMKII) phosphorylates Raf-1 at serine 338 and mediates Ras-stimulated Raf-1 activation. Cell Cycle.

[182] Baljuls A., Schmitz W., Mueller T., Zahedi R.P., Sickmann A., Hekman M., Rapp U.R. (2008). Positive Regulation of A-RAF by Phosphorylation of Isoform-specific Hinge Segment and Identification of Novel Phosphorylation Sites. J. Biol. Chem..

[183] Zhang B.-H., Guan K.-L. (2000). Activation of B-Raf kinase requires phosphorylation of the conserved residues Thr598 and Ser601. EMBO J..

[184] Chong H., Lee J., Guan K. (2001). Positive and negative regulation of Raf kinase activity and function by phosphorylation. EMBO J..

[185] Köhler M., Röring M., Schorch B., Heilmann K., Stickel N., Fiala G.J., Schmitt L.C., Braun S., Ehrenfeld S., Uhl F.M. (2016). Activation loop phosphorylation regulates B-Raf in vivo and transformation by B-Raf mutants. EMBO J..

[186] Köhler M., Brummer T. (2016). B-Raf activation loop phosphorylation revisited. Cell Cycle.

[187] Varga A., Baccarini M. (2016). Knock-in(g) RAF for a loop. EMBO J..

[188] Grammatikakis N., Lin J., Grammatikakis A., Tsichlis P.N., Cochran B.H. (1999). p50 cdc37 Acting in Concert with Hsp90 Is Required for Raf-1 Function. Mol. Cell. Biol..

[189] Da Rocha Dias S., Friedlos F., Light Y., Springer C., Workman P., Marais R. (2005). Activated B-RAF is an Hsp90 client protein that is targeted by the anticancer drug 17-allylamino-17-demethoxygeldanamycin. Cancer Res..

[190] Dent P., Reardon D.B., Morrison D.K., Sturgill T.W. (1995). Regulation of Raf-1 and Raf-1 mutants by Ras-dependent and Ras-independent mechanisms in vitro. Mol. Cell. Biol..

[191] Fantl W.J., Muslin A.J., Kikuchi A., Martin J.A., MacNicol A.M., Grosst R.W., Williams L.T. (1994). Activation of Raf-1 by 14-3-3 proteins. Nature.

[192] Freed E., Symons M., Macdonald S.G., McCormick F., Ruggieri R. (1994). Binding of 14-3-3 proteins to the protein kinase Raf and effects on its activation. Science.

[193] Fu H., Xia K., Pallas D.C., Cui C., Conroy K., Narsimhan R.P., Mamon H., Collier R.J., Roberts T.M. (1994). Interaction of the protein kinase Raf-1 with 14-3-3 proteins. Science.

[194] Mandal A.K., Lee P., Chen J.A., Nillegoda N., Heller A., DiStasio S., Oen H., Victor J., Nair D.M., Brodsky J.L. (2007). Cdc37 has distinct roles in protein kinase quality control that protect nascent chains from degradation and promote posttranslational maturation. J. Cell Biol..

[195] Diedrich B., Rigbolt K.T., Röring M., Herr R., Kaeser-Pebernard S., Gretzmeier C., Murphy R.F., Brummer T., Dengjel J. (2017). Discrete cytosolic macromolecular BRAF complexes exhibit distinct activities and composition. EMBO J..

[196] Hunter T., Poon R.Y.C. (1997). Cdc37: A protein kinase chaperone?. Trends Cell Biol..

[197] Grbovic O.M., Basso A.D., Sawai A., Ye Q., Friedlander P., Solit D., Rosen N. (2006). V600E B-Raf requires the Hsp90 chaperone for stability and is degraded in response to Hsp90 inhibitors. Proc. Natl. Acad. Sci. USA.

[198] Smyth T., Paraiso K.H.T., Hearn K., Rodriguez-Lopez A.M., Munck J.M., Haarberg H.E., Sondak V.K., Thompson N.T., Azab M., Lyons J.F. (2014). Inhibition of HSP90 by AT13387 Delays the Emergence of Resistance to BRAF Inhibitors and Overcomes Resistance to Dual BRAF and MEK Inhibition in Melanoma Models. Mol. Cancer Ther..

[199] Paraiso K.H.T., Haarberg H.E., Wood E., Rebecca V.W., Chen Y.A., Xiang Y., Ribas A., Lo R.S., Weber J.S., Sondak V.K. (2012). The HSP90 Inhibitor XL888 Overcomes BRAF Inhibitor Resistance Mediated through Diverse Mechanisms. Clin. Cancer Res..

[200] Sullivan R.J. (2018). Forestalling BRAF-Inhibitor Resistance in a Shocking Way. Clin. Cancer Res..

[201] Kornfeld K., Hom D.B., Horvitz H.R. (1995). The ksr-1 gene encodes a novel protein kinase involved in Ras-mediated signaling in C. elegans. Cell.

[202] Boudeau J., Miranda-Saavedra D., Barton G.J., Alessi D.R. (2006). Emerging roles of pseudokinases. Trends Cell Biol..

[203] Michaud N.R., Therrien M., Cacace A., Edsall L.C., Spiegel S., Rubin G.M., Morrison D.K. (1997). KSR stimulates Raf-1 activity in a kinase-independent manner. Proc. Natl. Acad. Sci. USA.

[204] Douziech M., Sahmi M., Laberge G., Therrien M. (2006). A KSR/CNK complex mediated by HYP, a novel SAM domain-containing protein, regulates RAS-dependent RAF activation in Drosophila. Genes Dev..

[205] Sundaram M., Han M., The C. (1995). elegans ksr-1 gene encodes a novel raf-related kinase involved in Ras-mediated signal transduction. Cell.

[206] Therrien M., Chang H.C., Solomon N.M., Karim F.D., Wassarman D.A., Rubin G.M. (1995). KSR, a novel protein kinase required for RAS signal transduction. Cell.

[207] Lavoie H., Sahmi M., Maisonneuve P., Marullo S.A., Thevakumaran N., Jin T., Kurinov I., Sicheri F., Therrien M. (2018). MEK drives BRAF activation through allosteric control of KSR proteins. Nature.

[208] Brennan D.F., Dar A.C., Hertz N.T., Chao W.C.H., Burlingame A.L., Shokat K.M., Barford D. (2011). A Raf-induced allosteric transition of KSR stimulates phosphorylation of MEK. Nature.

[209] Pennington K.L., Chan T.Y., Torres M.P., Andersen J.L. (2018). The dynamic and stress-adaptive signaling hub of 14-3-3: Emerging mechanisms of regulation and context-dependent protein–protein interactions. Oncogene.

[210] Yaffe M.B. (2002). How do 14-3-3 proteins work?—Gatekeeper phosphorylation and the molecular anvil hypothesis. FEBS Lett..

[211] Freeman A.K., Morrison D.K. (2011). 14-3-3 Proteins: Diverse functions in cell proliferation and cancer progression. Semin. Cell Dev. Biol..

[212] Mischak H., Seitz T., Janosch P., Eulitz M., Steen H., Schellerer M., Philipp A., Kolch W. (1996). Negative regulation of Raf-1 by phosphorylation of serine 621. Mol. Cell. Biol..

[213] Park E., Rawson S., Li K., Kim B.-W., Ficarro S.B., Pino G.G.-D., Sharif H., Marto J.A., Jeon H., Eck M.J. (2019). Architecture of autoinhibited and active BRAF–MEK1–14-3-3 complexes. Nature.

[214] Cook S.J., McCormick F. (1993). Inhibition by cAMP of Ras-dependent activation of Raf. Science.

[215] Dhillon A.S., Pollock C., Steen H., Shaw P.E., Mischak H., Kolch W. (2002). Cyclic AMP-dependent kinase regulates Raf-1 kinase mainly by phosphorylation of serine 259. Mol. Cell. Biol..

[216] Wu J., Dent P., Jelinek T., Wolfman A., Weber M.J., Sturgill T.W. (1993). Inhibition of the EGF-activated MAP kinase signaling pathway by adenosine 3’,5’-monophosphate. Science.

[217] Zimmermann S., Moelling K. (1999). Phosphorylation and Regulation of Raf by Akt (Protein Kinase B). Science.

[218] Rommel C., Clarke B.A., Zimmermann S., Nuñez L., Rossman R., Reid K., Moelling K., Yancopoulos G.D., Glass D.J. (1999). Differentiation Stage-Specific Inhibition of the Raf-MEK-ERK Pathway by Akt. Science.

[219] Yuan J., Ng W.H., Yap J., Chia B., Huang X., Wang M., Hu J. (2018). The AMPK inhibitor overcomes the paradoxical effect of RAF inhibitors through blocking phospho–Ser-621 in the C terminus of CRAF. J. Biol. Chem..

[220] Sprenkle A.B., Davies S.P., Carling D., Hardie D.G., Sturgill T.W. (1997). Identification of Raf-1 Ser621 kinase activity from NIH 3T3 cells as AMP-activated protein kinase. FEBS Lett..

[221] Romano D., Nguyen L.K., Matallanas D., Halasz M., Doherty C., Kholodenko B.N., Kolch W. (2014). Protein interaction switches coordinate Raf-1 and MST2/Hippo signalling. Nat. Cell Biol..

[222] Yuan J., Ng W.H., Tian Z., Yap J., Baccarini M., Chen Z., Hu J. (2018). Activating mutations in MEK1 enhance homodimerization and promote tumorigenesis. Sci. Signal..

[223] Röring M., Herr R., Fiala G.J., Heilmann K., Braun S., Eisenhardt A.E., Halbach S., Capper D., von Deimling A., Schamel W.W. (2012). Distinct requirement for an intact dimer interface in wild-type, V600E and kinase-dead B-Raf signalling. EMBO J..

[224] Dent P., Haser W., Haystead T.A., Vincent L.A., Roberts T.M., Sturgill T.W. (1992). Activation of mitogen-activated protein kinase kinase by v-Raf in NIH 3T3 cells and in vitro. Science.

[225] Haystead C.M.M., Wu J., Gregory P., Sturgill T.W., Haystead T.A.J. (1993). Functional expression of a MAP kinase kinase in COS cells and recognition by an anti-STE7/byrl antibody. FEBS Lett..

[226] Papin C., Denouel A., Calothy G., Eychène A. (1996). Identification of signalling proteins interacting with B-Raf in the yeast two-hybrid system. Oncogene.

[227] Papin C., Eychne A., Brunet A., Pages G., Pouyssegur J., Calothy G., Barnier J.V. (1995). B-Raf protein isoforms interact with and phosphorylate Mek-1 on serine. Oncogene.

[228] Corbalan-Garcia S., Yang S.S., Degenhardt K.R., Bar-Sagi D. (1996). Identification of the mitogen-activated protein kinase phosphorylation sites on human Sos1 that regulate interaction with Grb2. Mol. Cell. Biol..

[229] Saha M., Carriere A., Cheerathodi M., Zhang X., Lavoie G., Rush J., Roux P.P., Ballif B.A. (2012). RSK phosphorylates SOS1 creating 14-3-3-docking sites and negatively regulating MAPK activation. Biochem. J..

[230] Brummer T., Naegele H., Reth M., Misawa Y. (2003). Identification of novel ERK-mediated feedback phosphorylation sites at the C-terminus of B-Raf. Oncogene.

[231] Dougherty M.K., Müller J., Ritt D.A., Zhou M., Zhou X.Z., Copeland T.D., Conrads T.P., Veenstra T.D., Lu K.P., Morrison D.K. (2005). Regulation of Raf-1 by Direct Feedback Phosphorylation. Mol. Cell.

[232] Ritt D.A., Monson D.M., Specht S.I., Morrison D.K. (2010). Impact of Feedback Phosphorylation and Raf Heterodimerization on Normal and Mutant B-Raf Signaling. Mol. Cell. Biol..

[233] Ritt D.A., Abreu-Blanco M.T., Bindu L., Durrant D.E., Zhou M., Specht S.I., Stephen A.G., Holderfield M., Morrison D.K., Abreu-blanco T. (2016). Inhibition of Ras/Raf/MEK/ERK Pathway Signaling by a Stress-Induced Phospho-Regulatory Circuit. Mol. Cell.

[234] Holderfield M., Merritt H., Chan J., Wallroth M., Tandeske L., Zhai H., Tellew J., Hardy S., Hekmat-Nejad M., Stuart D.D. (2013). RAF Inhibitors Activate the MAPK Pathway by Relieving Inhibitory Autophosphorylation. Cancer Cell.

[235] Tassin T.C., Benavides D.R., Plattner F., Nishi A., Bibb J.A. (2015). Regulation of ERK kinase by MEK1 kinase inhibition in the brain. J. Biol. Chem..

[236] Von Kriegsheim A., Pitt A., Grindlay G.J., Kolch W., Dhillon A.S. (2006). Regulation of the Raf-MEK-ERK pathway by protein phosphatase 5. Nat. Cell Biol..

[237] Huang C.Y., Tan T.H. (2012). DUSPs, to MAP kinases and beyond. Cell Biosci..

[238] Zhang Z., Kobayashi S., Borczuk A.C., Leidner R.S., LaFramboise T., Levine A.D., Halmos B. (2010). Dual specificity phosphatase 6 (DUSP6) is an ETS-regulated negative feedback mediator of oncogenic ERK signaling in lung cancer cells. Carcinogenesis.

[239] Ekerot M., Stavridis M.P., Delavaine L., Mitchell M.P., Staples C., Owens D.M., Keenan I.D., Dickinson R.J., Storey K.G., Keyse S.M. (2008). Negative-feedback regulation of FGF signalling by DUSP6/MKP-3 is driven by ERK1/2 and mediated by Ets factor binding to a conserved site within the DUSP6/MKP-3 gene promoter. Biochem. J..

[240] Hacohen N., Kramer S., Sutherland D., Hiromi Y., Krasnow M.A. (1998). sprouty Encodes a Novel Antagonist of FGF Signaling that Patterns Apical Branching of the Drosophila Airways. Cell.

[241] Yusoff P., Lao D.-H., Ong S.H., Wong E.S.M., Lim J., Lo T.L., Leong H.F., Fong C.W., Guy G.R. (2002). Sprouty2 Inhibits the Ras/MAP Kinase Pathway by Inhibiting the Activation of Raf. J. Biol. Chem..

[242] Sasaki A., Taketomi T., Kato R., Saeki K., Nonami A., Sasaki M., Kuriyama M., Saito N., Shibuya M., Yoshimura A. (2003). Mammalian Sprouty4 suppresses Ras-independent ERK activation by binding to Raf1. Nat. Cell Biol..

[243] Simanshu D.K., Nissley D.V., McCormick F. (2017). RAS Proteins and Their Regulators in Human Disease. Cell.

[244] Haigis K.M., Kendall K.R., Wang Y., Cheung A., Haigis M.C., Glickman J.N., Niwa-Kawakita M., Sweet-Cordero A., Sebolt-Leopold J., Shannon K.M. (2008). Differential effects of oncogenic K-Ras and N-Ras on proliferation, differentiation and tumor progression in the colon. Nat. Genet..

[245] Drosten M., Simón-Carrasco L., Hernández-Porras I., Lechuga C.G., Blasco M.T., Jacob H.K.C., Fabbiano S., Potenza N., Bustelo X.R., Guerra C. (2017). H-Ras and K-Ras Oncoproteins Induce Different Tumor Spectra When Driven by the Same Regulatory Sequences. Cancer Res..

[246] Scheffzek K., Ahmadian M.R., Kabsch W., Wiesmüller L., Lautwein A., Schmitz F., Wittinghofer A. (1997). The Ras-RasGAP complex: Structural basis for GTPase activation and its loss in oncogenic ras mutants. Science.

[247] Rabara D., Tran T.H., Dharmaiah S., Stephens R.M., McCormick F., Simanshu D.K., Holderfield M. (2019). KRAS G13D sensitivity to neurofibromin-mediated GTP hydrolysis. Proc. Natl. Acad. Sci. USA.

[248] Gremer L., Gilsbach B., Reza Ahmadian M., Wittinghofer A. (2008). Fluoride complexes of oncogenic Ras mutants to study the Ras-RasGAP interaction. Biol. Chem..

[249] Fukushima T., Suzuki S., Mashiko M., Ohtake T., Endo Y., Takebayashi Y., Sekikawa K., Hagiwara K., Takenoshita S. (2003). BRAF mutations in papillary carcinomas of the thyroid. Oncogene.

[250] Forbes S.A., Bindal N., Bamford S., Cole C., Kok C.Y., Beare D., Jia M., Shepherd R., Leung K., Menzies A. (2010). COSMIC: Mining complete cancer genomes in the Catalogue of Somatic Mutations in Cancer. Nucl. Acids Res..

[251] Flaherty K.T., McArthur G. (2010). BRAF, a target in melanoma. Cancer.

[252] Wan P.T., Garnett M.J., Roe S.M., Lee S., Niculescu-Duvaz D., Good V.M., Project C.G., Jones C.M., Marshall C.J., Springer C.J. (2004). Mechanism of Activation of the RAF-ERK Signaling Pathway by Oncogenic Mutations of B-RAF. Cell.

[253] Lavoie H., Therrien M. (2015). Regulation of RAF protein kinases in ERK signalling. Nat. Rev. Mol. Cell Biol..

[254] Ikenoue T., Hikiba Y., Kanai F., Tanaka Y., Imamura J., Imamura T., Ohta M., Ijichi H., Tateishi K., Kawakami T. (2003). Functional Analysis of Mutations within the Kinase Activation Segment of B-Raf in Human Colorectal Tumors. Cancer Res..

[255] Andreadi C., Cheung L.-K., Giblett S., Patel B., Jin H., Mercer K., Kamata T., Lee P., Williams A., McMahon M. (2012). The intermediate-activity (L597V)BRAF mutant acts as an epistatic modifier of oncogenic RAS by enhancing signaling through the RAF/MEK/ERK pathway. Genes Dev..

[256] Dahlman K.B., Xia J., Hutchinson K., Ng C., Hucks D., Jia P., Atefi M., Su Z., Branch S., Lyle P.L. (2012). BRAF L597 Mutations in Melanoma Are Associated with Sensitivity to MEK Inhibitors. Cancer Discov..

[257] Houben R., Becker J., Kappel A., Terheyden P., Brocker E.-B., Goetz R., Rapp U. (2004). Constitutive activation of the Ras-Raf signaling pathway in metastatic melanoma is associated with poor prognosis. J. Carcinog..

[258] Ikenoue T., Hikiba Y., Kanai F., Aragaki J., Tanaka Y., Imamura J., Imamura T., Ohta M., Ijichi H., Tateishi K. (2004). Different Effects of Point Mutations within the B-Raf Glycine-Rich Loop in Colorectal Tumors on Mitogen-Activated Protein/Extracellular Signal-Regulated Kinase Kinase/Extracellular Signal-Regulated Kinase and Nuclear Factor κB Pathway and Cellular Transfo. Cancer Res..

[259] Noeparast A., Teugels E., Giron P., Verschelden G., Brakeleer S. De, Decoster L., Grève J. (2016). De Non-V600 BRAF mutations recurrently found in lung cancer predict sensitivity to the combination of Trametinib and Dabrafenib. Oncotarget.

[260] Swofford B.P., Homsi J. (2017). Uncommon BRAF Mutations Associated with Durable Response to Immunotherapy in Patients with Metastatic Melanoma. Case Rep. Oncol. Med..

[261] Smalley K.S.M., Xiao M., Villanueva J., Nguyen T.K., Flaherty K.T., Letrero R., Van Belle P., Elder D.E., Wang Y., Nathanson K.L. (2009). CRAF inhibition induces apoptosis in melanoma cells with non-V600E BRAF mutations. Oncogene.

[262] Yao Z., Torres N.M., Tao A., Gao Y., Luo L., Li Q., de Stanchina E., Abdel-Wahab O., Solit D.B., Poulikakos P.I. (2015). BRAF Mutants Evade ERK-Dependent Feedback by Different Mechanisms that Determine Their Sensitivity to Pharmacologic Inhibition. Cancer Cell.

[263] Jones D.T.W., Kocialkowski S., Liu L., Pearson D.M., Bäcklund L.M., Ichimura K., Collins V.P. (2008). Tandem Duplication Producing a Novel Oncogenic BRAF Fusion Gene Defines the Majority of Pilocytic Astrocytomas. Cancer Res..

[264] Cin H., Meyer C., Herr R., Janzarik W.G., Lambert S., Jones D.T.W., Jacob K., Benner A., Witt H., Remke M. (2011). Oncogenic FAM131B–BRAF fusion resulting from 7q34 deletion comprises an alternative mechanism of MAPK pathway activation in pilocytic astrocytoma. Acta Neuropathol..

[265] Ciampi R., Knauf J.A., Kerler R., Gandhi M., Zhu Z., Nikiforova M.N., Rabes H.M., Fagin J.A., Nikiforov Y.E. (2005). Oncogenic AKAP9-BRAF fusion is a novel mechanism of MAPK pathway activation in thyroid cancer. J. Clin. Investig..

[266] Zehir A., Benayed R., Shah R.H., Syed A., Middha S., Kim H.R., Srinivasan P., Gao J., Chakravarty D., Devlin S.M. (2017). Mutational landscape of metastatic cancer revealed from prospective clinical sequencing of 10,000 patients. Nat. Med..

[267] Sarkozy A., Carta C., Moretti S., Zampino G., Digilio M.C., Pantaleoni F., Scioletti A.P., Esposito G., Cordeddu V., Lepri F. (2009). Germline BRAF mutations in Noonan, LEOPARD, and cardiofaciocutaneous syndromes: Molecular diversity and associated phenotypic spectrum. Hum. Mutat..

[268] Travers T., López C.A., Van Q.N., Neale C., Tonelli M., Stephen A.G., Gnanakaran S. (2018). Molecular recognition of RAS/RAF complex at the membrane: Role of RAF cysteine-rich domain. Sci. Rep..

[269] Fetics S.K., Guterres H., Kearney B.M., Buhrman G., Ma B., Nussinov R., Mattos C. (2015). Allosteric effects of the oncogenic rasq61l mutant on raf-RBD. Structure.

[270] Leicht D.T., Balan V., Kaplun A., Singh-Gupta V., Kaplun L., Dobson M., Tzivion G. (2007). Raf kinases: Function, regulation and role in human cancer. Biochim. Biophys. Acta Mol. Cell Res..

[271] Molzan M., Schumacher B., Ottmann C., Baljuls A., Polzien L., Weyand M., Thiel P., Rose R., Rose M., Kuhenne P. (2010). Impaired Binding of 14-3-3 to C-RAF in Noonan Syndrome Suggests New Approaches in Diseases with Increased Ras Signaling. Mol. Cell. Biol..

[272] Brown N.A., Furtado L.V., Betz B.L., Kiel M.J., Weigelin H.C., Lim M.S., Elenitoba-Johnson K.S.J. (2014). High prevalence of somatic MAK1 mutations in BRAF V600E–negative Langerhans cell histiocytosis. Blood.

[273] Brenan L., Andreev A., Cohen O., Pantel S., Kamburov A., Cacchiarelli D., Persky N.S., Zhu C., Bagul M., Goetz E.M. (2016). Phenotypic Characterization of a Comprehensive Set of MAPK1/ERK2 Missense Mutants. Cell Rep..

[274] Cox A.D., Fesik S.W., Kimmelman A.C., Luo J., Der C.J. (2014). Drugging the undruggable RAS: Mission Possible?. Nat. Rev. Drug Discov..

[275] Ostrem J.M., Peters U., Sos M.L., Wells J.A., Shokat K.M. (2013). K-Ras(G12C) inhibitors allosterically control GTP affinity and effector interactions. Nature.

[276] Fakih M., O’Neil B., Price T.J., Falchook G.S., Desai J., Kuo J., Govindan R., Rasmussen E., Morrow P.K.H., Ngang J. (2019). Phase 1 study evaluating the safety, tolerability, pharmacokinetics (PK), and efficacy of AMG 510, a novel small molecule KRASG12C inhibitor, in advanced solid tumors. J. Clin. Oncol..

[277] Christensen J.G., Fell J.B., Marx M.A., Fischer J., Hallin J., Calinisan A., Baer B., Burkhard M., Blake J., Vigers G. (2019). Abstract LB-271: Insight towards therapeutic susceptibility of KRAS mutant cancers from MRTX1257, a novel KRAS G12C mutant selective small molecule inhibitor. Cancer Res..

[278] Zeitouni D., Pylayeva-Gupta Y., Der C.J., Bryant K.L. (2016). KRAS Mutant Pancreatic Cancer: No Lone Path to an Effective Treatment. Cancers.

[279] Gibbs J.B., Oliff A., Kohl N.E. (1994). Farnesyltransferase inhibitors: Ras research yields a potential cancer therapeutic. Cell.

[280] Furfine E.S., Leban J.J., Landavazo A., Moomaw J.F., Casey P.J. (1995). Protein farnesyltransferase: Kinetics of farnesyl pyrophosphate binding and product release. Biochemistry.

[281] Long S.B., Casey P.J., Beese L.S. (1998). Cocrystal Structure of Protein Farnesyltransferase Complexed with a Farnesyl Diphosphate Substrate. Biochemistry.

[282] Long S.B., Casey P.J., Beese L.S. (2002). Reaction path of protein farnesyltransferase at atomic resolution. Nature.

[283] Mijimolle N., Velasco J., Dubus P., Guerra C., Weinbaum C.A., Casey P.J., Campuzano V., Barbacid M. (2005). Protein farnesyltransferase in embryogenesis, adult homeostasis, and tumor development. Cancer Cell.

[284] Whyte D.B., Kirschmeier P., Hockenberry T.N., Nunez-Oliva I., James L., Catino J.J., Bishop W.R., Pai J.-K. (1997). K- and N-Ras Are Geranylgeranylated in Cells Treated with Farnesyl Protein Transferase Inhibitors. J. Biol. Chem..

[285] Fiordalisi J.J., Johnson R.L., Weinbaum C.A., Sakabe K., Chen Z., Casey P.J., Cox A.D. (2003). High affinity for farnesyltransferase and alternative prenylation contribute individually to K-Ras4B resistance to farnesyltransferase inhibitors. J. Biol. Chem..

[286] Berndt N., Hamilton A.D., Sebti S.M. (2011). Targeting protein prenylation for cancer therapy. Nat. Rev. Cancer.

[287] Peterson Y.K., Kelly P., Weinbaum C.A., Casey P.J. (2006). A Novel Protein Geranylgeranyltransferase-I Inhibitor with High Potency, Selectivity, and Cellular Activity. J. Biol. Chem..

[288] Sjogren A.-K.M., Andersson K.M.E., Liu M., Cutts B.A., Karlsson C., Wahlstrom A.M., Dalin M., Weinbaum C., Casey P.J., Tarkowski A. (2007). GGTase-I deficiency reduces tumor formation and improves survival in mice with K-RAS–induced lung cancer. J. Clin. Investig..

[289] Lobell R.B., Liu D., Buser C.A., Davide J.P., DePuy E., Hamilton K., Koblan K.S., Lee Y., Mosser S., Motzel S.L. (2002). Preclinical and Clinical Pharmacodynamic Assessment of L-778,123, a Dual Inhibitor of Farnesyl:Protein Transferase and Geranylgeranyl: Protein Transferase Type-I. Mol. Cancer Ther..

[290] Wahlstrom A.M., Cutts B.A., Liu M., Lindskog A., Karlsson C., Sjogren A.-K.M., Andersson K.M.E., Young S.G., Bergo M.O. (2008). Inactivating Icmt ameliorates K-RAS–induced myeloproliferative disease. Blood.

[291] Bergman J.A., Hahne K., Song J., Hrycyna C.A., Gibbs R.A. (2012). S-Farnesyl-Thiopropionic Acid Triazoles as Potent Inhibitors of Isoprenylcysteine Carboxyl Methyltransferase. ACS Med. Chem. Lett..

[292] Lau H.Y., Ramanujulu P.M., Guo D., Yang T., Wirawan M., Casey P.J., Go M.-L., Wang M. (2014). An improved isoprenylcysteine carboxylmethyltransferase inhibitor induces cancer cell death and attenuates tumor growth in vivo. Cancer Biol. Ther..

[293] Wang M., Hossain M.S., Tan W., Coolman B., Zhou J., Liu S., Casey P.J. (2010). Inhibition of isoprenylcysteine carboxylmethyltransferase induces autophagic-dependent apoptosis and impairs tumor growth. Oncogene.

[294] Athuluri-Divakar S.K., Vasquez-Del Carpio R., Dutta K., Baker S.J., Cosenza S.C., Basu I., Gupta Y.K., Reddy M.V.R., Ueno L., Hart J.R. (2016). A Small Molecule RAS-Mimetic Disrupts RAS Association with Effector Proteins to Block Signaling. Cell.

[295] Keeton A.B., Salter E.A., Piazza G.A. (2017). The RAS–Effector Interaction as a Drug Target. Cancer Res..

[296] Kato-Stankiewicz J., Hakimi I., Zhi G., Zhang J., Serebriiskii I., Guo L., Edamatsu H., Koide H., Menon S., Eckl R. (2002). Inhibitors of Ras/Raf-1 interaction identified by two-hybrid screening revert Ras-dependent transformation phenotypes in human cancer cells. Proc. Natl. Acad. Sci. USA.

[297] Wang W., Fang G., Rudolph J. (2012). Ras inhibition via direct Ras binding—Is there a path forward?. Bioorg. Med. Chem. Lett..

[298] Bryant K.L., Stalnecker C.A., Zeitouni D., Klomp J.E., Peng S., Tikunov A.P., Gunda V., Pierobon M., Waters A.M., George S.D. (2019). Combination of ERK and autophagy inhibition as a treatment approach for pancreatic cancer. Nat. Med..

[299] Kinsey C.G., Camolotto S.A., Boespflug A.M., Guillen K.P., Foth M., Truong A., Schuman S.S., Shea J.E., Seipp M.T., Yap J.T. (2019). Protective autophagy elicited by RAF→MEK→ERK inhibition suggests a treatment strategy for RAS-driven cancers. Nat. Med..

[300] Wang M., Tan W., Zhou J., Leow J., Go M., Lee H.S., Casey P.J. (2008). A Small Molecule Inhibitor of Isoprenylcysteine Carboxymethyltransferase Induces Autophagic Cell Death in PC3 Prostate Cancer Cells. J. Biol. Chem..

[301] Teh J.T., Zhu W.L., Ilkayeva O.R., Li Y., Gooding J., Casey P.J., Summers S.A., Newgard C.B., Wang M. (2015). Isoprenylcysteine carboxylmethyltransferase regulates mitochondrial respiration and cancer cell metabolism. Oncogene.

[302] Manu K.A., Chai T.F., Teh J.T., Zhu W.L., Casey P.J., Wang M. (2017). Inhibition of Isoprenylcysteine Carboxylmethyltransferase Induces Cell-Cycle Arrest and Apoptosis through p21 and p21-Regulated BNIP3 Induction in Pancreatic Cancer. Mol. Cancer Ther..

[303] Thompson N., Adams D.J., Ranzani M. (2017). Synthetic lethality: Emerging targets and opportunities in melanoma. Pigment Cell Melanoma Res..

[304] Shen J.P., Zhao D., Sasik R., Luebeck J., Birmingham A., Bojorquez-Gomez A., Licon K., Klepper K., Pekin D., Beckett A.N. (2017). Combinatorial CRISPR-Cas9 screens for de novo mapping of genetic interactions. Nat. Methods.

[305] Flaherty K., Puzanov I., Sosman J., Kim K., Ribas A., McArthur G., Lee R.J., Grippo J.F., Nolop K., Chapman P. (2009). Phase I study of PLX4032: Proof of concept for V600E BRAF mutation as a therapeutic target in human cancer. J. Clin. Oncol..

[306] Tsai J., Lee J.T., Wang W., Zhang J., Cho H., Mamo S., Bremer R., Gillette S., Kong J., Haass N.K. (2008). Discovery of a selective inhibitor of oncogenic B-Raf kinase with potent antimelanoma activity. Proc. Natl. Acad. Sci. USA.

[307] Hauschild A., Grob J.-J., Demidov L.V., Jouary T., Gutzmer R., Millward M., Rutkowski P., Blank C.U., Miller W.H., Kaempgen E. (2012). Dabrafenib in BRAF mutated metastatic melanoma: A multicentre, open-label, phase 3 randomised controlled trial. Lancet.

[308] Li Z., Jiang K., Zhu X., Lin G., Song F., Zhao Y., Piao Y., Liu J., Cheng W., Bi X. (2016). Encorafenib (LGX818), a potent BRAF inhibitor, induces senescence accompanied by autophagy in BRAFV600E melanoma cells. Cancer Lett..

[309] Wang L., Leite de Oliveira R., Huijberts S., Bosdriesz E., Pencheva N., Brunen D., Bosma A., Song J.Y., Zevenhoven J., Los-de Vries G.T. (2018). An Acquired Vulnerability of Drug-Resistant Melanoma with Therapeutic Potential. Cell.

[310] Seghers A.C., Wilgenhof S., Lebbé C., Neyns B. (2012). Successful rechallenge in two patients with BRAF-V600-mutant melanoma who experienced previous progression during treatment with a selective BRAF inhibitor. Melanoma Res..

[311] Peng S.-B., Henry J.R., Kaufman M.D., Lu W.-P., Smith B.D., Vogeti S., Rutkoski T.J., Wise S., Chun L., Zhang Y. (2015). Inhibition of RAF Isoforms and Active Dimers by LY3009120 Leads to Anti-tumor Activities in RAS or BRAF Mutant Cancers. Cancer Cell.

[312] Okaniwa M., Hirose M., Arita T., Yabuki M., Nakamura A., Takagi T., Kawamoto T., Uchiyama N., Sumita A., Tsutsumi S. (2013). Discovery of a Selective Kinase Inhibitor (TAK-632) Targeting Pan-RAF Inhibition: Design, Synthesis, and Biological Evaluation of C-7-Substituted 1,3-Benzothiazole Derivatives. J. Med. Chem..

[313] Sun Y., Alberta J.A., Pilarz C., Calligaris D., Chadwick E.J., Ramkissoon S.H., Ramkissoon L.A., Garcia V.M., Mazzola E., Goumnerova L. (2017). A brain-penetrant RAF dimer antagonist for the noncanonical BRAF oncoprotein of pediatric low-grade astrocytomas. Neuro. Oncol..

[314] Dean E.J., Banerji U., Girotti R., Niculescu-Duvaz I., Lopes F., Davies L., Niculescu-Duvaz D., Dhomen N., Ellis S., Ali Z. (2016). A Phase 1 first-in-human trial to evaluate the safety and tolerability of CCT3833, an oral panRAF inhibitor, in patients with advanced solid tumours, including metastatic melanoma. J. Clin. Oncol..

[315] Saturno G., Lopes F., Girotti M.R., Niculescu-Duvaz I., Niculescu-Duvaz D., Zambon A., Davies L., Johnson L., Preece N., Viros A. (2016). Therapeutic efficacy of the paradox-breaking panRAF and SRC drug CCT3833/BAL3833 in KRAS-driven cancer models. Eur. J. Cancer.

[316] Ramurthy S., Taft B.R., Aversa R.J., Barsanti P.A., Burger M.T., Lou Y., Nishiguchi G.A., Rico A., Setti L., Smith A. (2019). Design and Discovery of N-(3-(2-(2-Hydroxyethoxy)-6-morpholinopyridin-4-yl)-4-methylphenyl)-2-(trifluoromethyl)isonicotinamide, a Selective, Efficacious, and Well-Tolerated RAF Inhibitor Targeting RAS Mutant Cancers: The Path to the Clinic. J. Med. Chem..

[317] Williams T.E., Subramanian S., Verhagen J., McBride C.M., Costales A., Sung L., Antonios-McCrea W., McKenna M., Louie A.K., Ramurthy S. (2015). Discovery of RAF265: A Potent mut-B-RAF Inhibitor for the Treatment of Metastatic Melanoma. ACS Med. Chem. Lett..

[318] Yao Z., Gao Y., Su W., Yaeger R., Tao J., Na N., Zhang Y., Zhang C., Rymar A., Tao A. (2019). RAF inhibitor PLX8394 selectively disrupts BRAF dimers and RAS-independent BRAF-mutant-driven signaling. Nat. Med..

[319] Tutuka C.S.A., Andrews M.C., Mariadason J.M., Ioannidis P., Hudson C., Cebon J., Behren A. (2017). PLX8394, a new generation BRAF inhibitor, selectively inhibits BRAF in colonic adenocarcinoma cells and prevents paradoxical MAPK pathway activation. Mol. Cancer.

[320] Zhang C., Spevak W., Zhang Y., Burton E.A., Ma Y., Habets G., Zhang J., Lin J., Ewing T., Matusow B. (2015). RAF inhibitors that evade paradoxical MAPK pathway activation. Nature.

[321] Cheng Y., Tian H. (2017). Current development status of MEK inhibitors. Molecules.

[322] Welsh S.J., Corrie P.G. (2015). Management of BRAF and MEK inhibitor toxicities in patients with metastatic melanoma. Ther. Adv. Med. Oncol..

[323] Wang P.-F., Qiu H.-Y., Zhu H.-L. (2019). A patent review of BRAF inhibitors: 2013–2018. Expert Opin. Ther. Pat..

[324] Molina-Arcas M., Downward J. (2012). How to fool a wonder drug: Truncate and dimerize. Cancer Cell.

